# Evolution-Informed Viroinformatics Identifies Conserved Immune Targets with Predicted Lower Mutational Vulnerability Across Betacoronaviruses

**DOI:** 10.3390/v18101171

**Published:** 2026-10-21

**Authors:** Jamilah Alshammari, Huda A. Alqahtani, Rasha Alonaizan, Inam ur Rahman, Khaled Alzhrani, Nawal Al-Hoshani, Maha Abdullah Alwaili, Tariq Aziz

**Affiliations:** 1 Department of Zoology, College of Science, King Saud University, Riyadh 11495, Saudi Arabia; 2 Fuli Institute of Food Science, College of Biosystems Engineering and Food Science, Zhejiang University, Hangzhou 310058, China; 3 Department of Clinical Laboratory Sciences, College of Applied Medical Sciences, Jouf University, Sakaka 72388, Saudi Arabia; 4 Department of Biology, College of Science, Princess Nourah bint Abdulrahman University, P.O. Box 84428, Riyadh 11671, Saudi Arabia; 5 Department of Biology, College of Science, University of Tabuk, Tabuk 71491, Saudi Arabia; 6 Molecular Microbiology and Infectious Diseases Research Unit, University of Tabuk, Tabuk 71491, Saudi Arabia

**Keywords:** pan-betacoronavirus, multi-epitope vaccine, immunoinformatics, evolutionary conservation, mutational escape, reverse vaccinology

## Abstract

The extensive evolutionary diversity and continuous mutational adaptation of betacoronaviruses represent a major challenge to the development of broadly protective countermeasures. This study applied an evolution-informed Viroinformatics framework to systematically identify conserved and immune targets with predicted lower mutational vulnerability across betacoronaviruses. The workflow integrated comparative sequence conservation and phylogenetic analysis with immunoinformatics-based epitope screening, HLA population coverage, mutational escape profiling, structural modeling, molecular docking, and molecular dynamics simulations. Conserved cytotoxic T-lymphocyte and helper T-lymphocyte epitopes were prioritized based on immunogenicity, antigenicity, safety, HLA binding potential, evolutionary conservation, and predicted resistance to mutational escape. Combined MHC class I and II analysis predicted a theoretical global population coverage of 99.92%, which requires experimental validation. The resulting 409-amino-acid multiepitope construct was predicted to be antigenic, non-allergenic, and physiochemically stable. Structural validation showed that 99.7% of the residues were located within favored or additionally allowed regions. Molecular docking demonstrated favorable interaction of the vaccine construct with TLR7, while a 100 ns molecular dynamics simulation supported persistence of the predicted vaccine-receptor association. Overall, these findings provide a computational framework for prioritizing evolutionarily conserved immune targets and developing pre-emptive candidate epitopes showing predicted cross-betacoronavirus conservation within the analyzed dataset. Experimental studies evaluating HLA binding, cellular and humoral immunogenicity, recombinant expression, cross-reactivity, and in vivo protection are required to validate the predicted immunological potential and determine whether the proposed construct can provide broad protection against diverse betacoronaviruses.

## 1. Introduction

Betacoronaviruses constitute an evolutionarily diverse group of enveloped RNA viruses with a remarkable capacity for host adaptation, cross-species transmission, and genetic diversification. The emergence of severe acute respiratory syndrome coronavirus (SARS-CoV), Middle East respiratory syndrome coronavirus (MERS-CoV) and SARS CoV-2 has demonstrated the recurrent zoonotic potential of this viral lineage and highlighted the possibility that additional betacoronaviruses may emerge as future human pathogens [1]. Their high evolutionary plasticity, driven by mutation, recombination, and selection pressures, presents a major challenge for the development of countermeasures capable of retaining effectiveness across genetically diverse and continuously evolving viruses [2,3,4]. Current vaccine strategies have largely focused on individual viruses and, particularly for SARS CoV-2, immunodominant but evolutionarily variable regions of the spike protein. Although these approaches have provided substantial protection against severe disease, continued viral evolution can alter antigenic sites and facilitate immune escape. This has increased interest in identifying immune determinants that are conserved across divergent betacoronaviruses and remain comparatively resistant to mutational change. Such evolutionarily constrained regions may provide more durable targets for the development of broadly applicable or pre-emptive vaccine strategies [2,3,4].

While there are currently vaccines that are effective against certain viruses, the ability of viruses to evolve quickly and new coronavirus strains emerging are a reminder of the need for stronger and more forward-looking vaccine strategies [1,2,3]. Betacoronavirus refers to a genus of genetically and antigenically diverse viruses that are found in many animal reservoirs and in humans. They are closely related to SARS-CoV, MERS-CoV, and SARS-CoV-2 and also contain endemic viruses that occur in humans, viruses found in bats, pangolins, camels, and other mammals. This wide host range provides opportunities for inter-species transmission, viral adaptation, and the spread of new pathogenic types. Therefore, single virus preparedness efforts targeting the primary virus in circulation could offer protection against antigenically dissimilar betacoronaviruses found in animals [2,4].

The spike glycoprotein is a main target for vaccination against the coronavirus due to its essential function in binding host cells during entry and its immunogenicity. There are, however, highly variable and evolutionarily conserved areas in the spike proteins. Conserved regions, especially those that are important for structural and functional functions, can be useful targets for vaccine development to recognize more than just one strain of the virus that causes COVID-19. A combination of those areas and B-cell, CTL, and HTL epitope screening could allow for targeting both humoral and cellular immunity simultaneously [5,6,7]. In terms of function, the coronavirus spike protein has sequences that are more or less under selection pressure. The receptor-binding regions can have a considerable amount of sequence variation within them, but the structural regions and the membrane fusion regions are more likely to be under tight functional constraints, as mutations that affect them might result in loss of viral entry. These conserved elements are thus promising targets for widespread vaccine development. Targeting epitopes residing in structurally or evolutionarily constrained areas could alleviate the need for strain-specific antigenic sites and could increase recognition of genetically divergent betacoronavirus strains. But it is possible that the same sequence might not suffice, as individual conserved residues might be quite different in terms of tolerance to future substitutions [8].

An alternative approach is multi-epitope vaccine design, where multiple immune determinants are included in a single vaccine construct. B-cell epitopes can facilitate antibody-mediated recognition, while the MHC class I molecule-presented cytotoxic T-lymphocyte (CTL) epitopes can stimulate CD8+ T-cell responses to infected cells. Epitopes presented by MHC class II molecules can also be used to support cellular and humoral immune responses, called helper T-lymphocyte (HTL) epitopes [9]. The HLA molecules are very polymorphic across the human population, and introducing multiple alleles or HLA epitopes and assessing the population coverage are important points to consider when developing vaccine candidates for use in large and diverse geographical regions. Conserved B- and T-cell epitopes were previously identified from SARS-CoV, MERS-CoV, and SARS-CoV-2, and multi-epitope vaccine candidates were developed through the use of a combination of antigenicity, HLA-binding affinity, population coverage, allergenicity and toxicity, structural modeling, and molecular docking. The conserved viral regions have been increasingly studied as potential targets for more general protection in more recent pan-coronavirus research. These studies together prove the potential of computational approaches for vaccine design based on the immunogenic epitopes, but most of the proposed strategies are limited to a single coronavirus species or known circulating variants [10,11].

Overt changes in antigenic regions of the molecule can occur through mutation, selection, and recombination, leading to an eventual loss of recognition of the epitopes targeted by pre-existing immunity. The problem is especially important with regard to coronaviruses, as they have a history of high rates of genetic diversification and recombination. However, from the perspective of designing a vaccine, an epitope that is very conserved in the sequences that are currently sampled will not necessarily have the same resistance to evolutionary changes that it has to future evolutionary changes. The combination of entropy of epitopic sequences, residues to be conserved, and in silico mutational scanning may offer a more informative framework to identify conserved epitopes that are relatively less susceptible to possible substitutions associated with escape. Although these advances have been made, very little effort has been made to combine the conservation of cross-betacoronavirus epitopes with predicted evolutionary robustness. A conserved epitope may have residues that are vulnerable to changes in the future or immune escape. Moreover, traditional immunoinformatic pipelines typically don’t integrate phylogenetic diversity, residue-level conservation, entropy-based variability, mutational escape vulnerability, HLA population representation, and structural stability into a single preemptive vaccine-design pipeline. This is constraining their efforts to tackle the evolutionary uncertainty around potential future spillovers of coronaviruses [12].

Therefore, this study was conducted with the aim of developing an evolution-informed pan-betacoronavirus multi-epitope vaccine platform which encompasses conserved and potentially mutation-proof regions of the spike protein. A computational framework that combines sequence and phylogenetic analysis, conservation and diversity profiling, B-cell and T-cell epitope screening, HLA population coverage, cytokine prediction, mutational escape analysis, vaccine construction, structural validation, docking of immune receptors, and molecular dynamics simulation was used. The goal of this study was to identify vaccine determinants that may be relevant for a wide range of betacoronavirus species and to offer a computational basis for future experimental efforts in developing a universal coronavirus vaccine.

## 2. Materials and Methods

### 2.1. Retrieval and Curation of Betacoronavirus Spike Protein Sequences

Automated command-line data-fetching scripts were used to extract betacoronavirus spike glycoprotein sequences directly from the National Center for Biotechnology Information (NCBI) GenBank database (https://www.ncbi.nlm.nih.gov) for which the sequences were available, and the sequences were later manually curated for headers and multiple sequence aligned using progressive alignment algorithms to create the final standardized FASTA dataset (betacov_spike_aligned.fasta) [13].

### 2.2. Multiple Sequence Alignment

Multiple sequence alignment of curated spike protein sequences was performed using MAFFT (with the fast progressive algorithm FFT-NS-2) (https://www.ebi.ac.uk/jdispatcher/msa/mafft, Accessed on 13 July 2026) to create the positional homology among the different lines. The quality of the alignments was checked, and incorrect terminal gaps and low-confidence features were excluded. The residue-wise conservation scores were obtained by EMBOSS Cons (https://www.ebi.ac.uk/jdispatcher/msa/emboss_cons, Accessed on 13 July 2026), which is based on the frequencies of the amino acid properties, and highly conserved positions and stretches of conserved continuous structural motifs were defined by the standard threshold values [14,15].

### 2.3. Structural Superposition of Representative Spike Proteins

In order to compare the conformational conservation and spatial arrangement among the lineages, three-dimensional spike glycoprotein models were structurally superimposed on reference models with PyMOL 2.6 Molecular Graphics System (https://www.pymol.org). Using Align and Super, pairwise and global least-squares superposition was executed to obtain the residue-to-residue correspondence. Structural deviation was calculated as the root-mean-square deviation (RMSD), which was used to quantify structural stability and conformational homology of conserved functional domains [16].

### 2.4. Phylogenetic Analysis

The evolutionary relationship among aligned spike sequences of Betacoronavirus was estimated by IQ-TREE (https://iqtree.github.io, Accessed on 15 July 2026) with ModelFinder to automatically select the best substitution model, and using the ultrafast bootstrap approximation method (1000 replications) to determine the reliability of branch support. To map the divergence of clades, a taxonomic annotation by viral lineage and host group was performed. The resulting phylogenetic topology was analyzed to ensure that vaccine-targeted epitopes are localized in well-conserved functional nodes common to evolutionarily distant groups [17].

### 2.5. Pan-Betacoronavirus Conservation and Entropy Analysis

Conservation scores of each residue in the full multiple sequence alignment were calculated using Shannon entropy models with custom bioinformatics scripts, and sequence variability was quantified for each site in the alignment. Local sequence diversity was assessed in the spike glycoprotein using sliding-window entropy analyses (SWEA), over a window length equivalent to the epitopes. Regions with high entropy scores were recognized as regions tolerant to mutations in evolution and regions vulnerable to the processes of recombination, which were systematically excluded, and structural motifs conserved across all regions of the Betacoronavirus were prioritized for further exploitation in the downstream pan-Betacoronavirus epitope discovery [18].

### 2.6. Evolutionary Pressure and Recombination-Oriented Diversity Screening

Site-specific sequence variability and evolutionary pressure across the spike glycoprotein multiple sequence alignment were evaluated using sliding-window Shannon entropy analyses. Local diversity hotspots above the global thresholds for baseline entropy were identified to indicate areas with potentially high mutation rates. A systematic exclusion or deprioritization of these hypervariable segments in the process of epitope selection was made for pan-betacoronavirus cross-reactivity. The higher the entropy score, the more evolutionary mutational tolerance and recombination vulnerability, but not actual genetic recombination [19].

### 2.7. Extraction and Preliminary Immunological Profiling of Conserved Regions

Conserved spike-derived segments were then obtained from multi-sequence conservation profiles and were carefully put under preliminary immunological testing for safety and immunogenic efficacy. The intrinsic protective antigenicity was determined with the standard threshold parameter in VaxiJen v2.0 (https://www.ddg-pharmfac.net/vaxijen/VaxiJen/VaxiJen.html, Accessed on 17 July 2026) [20]. AllerTOP v2.1 (https://www.ddg-pharmfac.net/allertop_v2, Accessed on 17 July 2026) was then used to check the allergenicity of candidate sequences, and a search was conducted for potential toxicity. Only segments that proved to be highly antigenically active and that were strictly non-allergenic and non-toxic were carried forward to move downstream towards the discovery of multiple epitopes. Only those segments that were shown to be highly antigenic and that were strictly non-allergenic and non-toxic were advanced to go downstream towards multi-epitope discovery [21].

### 2.8. Linear B-Cell Epitope Prediction and Screening

The IEDB BepiPred Linear Epitope Prediction tool (https://tools.iedb.org/bcell, Accessed on 18 July 2026) was used to predict linear B-cell epitopes of conserved spike sequences, using a hidden Markov model that considers both hydrophobicity and secondary structure features [22]. Candidates within the set score were selected and passed a multi-parameter filter pipeline. The antigenicity was evaluated using VaxiJen v2.0 (https://www.ddg-pharmfac.net/vaxijen/VaxiJen/VaxiJen.html, Accessed on 19 July 2026), the allergenicity was evaluated using AllerTOP v2.1 (https://www.ddg-pharmfac.net/allertop_v2, Accessed on 19 July 2026), and the potential toxicity and the pan-betacoronavirus sequence conservancy were evaluated. High-confidence linear B-cell epitopes that met all the safety, antigenicity, and conservation requirements were only retained for downstream multi-epitope vaccine assembly [20,21].

### 2.9. CTL Epitope Prediction and MHC-I Binding Analysis

Conserved sequences of spike were used to predict the cytotoxic T-lymphocyte (CTL) epitopes by the IEDB MHC-I Binding Prediction tool (https://tools.iedb.org/mhci, Accessed on 20 July 2026) for the representative set of human leukocyte antigen (HLA) class I alleles [23]. Predicted half-maximal inhibitory concentration (IC50) values and percentile ranks were used to prioritize peptide-HLA binding interactions. Optimal candidates for vaccine construction were then filtered based on multi-parameter criteria that included antigenicity using VaxiJen v2.0 (https://www.ddg-pharmfac.net/vaxijen/VaxiJen/VaxiJen.html, Accessed on 20 July 2026), allergenicity using AllerTOP v2.1 (https://www.ddg-pharmfac.net/allertop_v2, Accessed on 21 July 2026), potential toxicity, and absolute sequence conservancy across all target betacoronavirus lineages [20,21].

### 2.10. HTL Epitope Prediction and MHC-II Binding Analysis

The conserved spike sequence was used to predict helper T-lymphocyte (HTL) epitopes using the IEDB MHC-II Binding Prediction tool (https://tools.iedb.org/mhcii, Accessed on 21 July 2026) against a representative panel of human leukocyte antigen (HLA) class II molecules that includes reference HLA-DR, HLA-DP, and HLA-DQ alleles. Consensus IC50 values and percentile rank thresholds were applied to calculate candidate peptide binding affinities and rank them [23]. The shortlisted HTL candidates were then screened for antigenicity using VaxiJen v2.0 (https://www.ddg-pharmfac.net/vaxijen/VaxiJen/VaxiJen.html, Accessed on 22 July 2026), allergenicity using AllerTOP v2.1 (https://www.ddg-pharmfac.net/allertop_v2, Accessed on 22 July 2026), potential toxicity, and pan-betacoronavirus sequence conservancy. High-affinity epitopes that passed all the immunological and safety criteria were selected for inclusion in the multi-epitope vaccine [20,21].

### 2.11. Population Coverage Analysis

The global and regional population coverage of the selected cytotoxic T-lymphocyte (CTL) and helper T-lymphocyte (HTL) epitopes were computed using the IEDB Population Coverage Analysis Tool (https://tools.iedb.org/population, Accessed on 23 July 2026), which relies on the known frequencies of human leukocyte antigen (HLA) alleles from different geographic regions around the world. The estimates for combined population coverage, average number of epitope/HLA hits per person, as well as the projected minimum coverage efficiency (PC90) were calculated. The most important final criterion was the broad population representation across all populations globally to obtain the broad-spectrum protective efficacy of the shortlisted candidate epitopes in all populations [24].

### 2.12. Cytokine-Induction Profiling

The predicted cytokine-inducing potential of the selected helper T-lymphocyte (HTL) and core multi-epitope candidates was assessed using IFNepitope (https://webs.iiitd.edu.in/raghava/ifnepitope/, Accessed on 24 July 2026), IL4pred (https://webs.iiitd.edu.in/raghava/il4pred/, Accessed on 24 July 2026) and IL10pred (https://webs.iiitd.edu.in/raghava/il10pred, Accessed on 25 July 2026). The capacity for inducing interferon gamma (IFN-γ), interleukin-4 (IL-4) and interleukin-10 (IL-10) was evaluated to describe their potential to stimulate Th1-mediated cellular immunity, Th2-associated immune responses and immunoregulatory activity, respectively. The cytokine induction profiles were then taken into account when prioritizing epitopes to find candidates with desirable predicted immunomodulatory properties [25,26,27].

### 2.13. Deep Mutational Scanning and Escape-Vulnerability Simulation

Single amino acid substitutions were systematically evaluated across the prioritized B-cell, MHC-I and MHC-II epitopes to see if they are likely to escape immunologically due to mutations. Vulnerability at each position was calculated by analyzing the predicted effect of the amino-acid substitutions on the stability of the epitope and on its potential for immune recognition. For each epitope, mutation-vulnerability profiles were used to recognize mutation hotspots from comparatively mutation-tolerant residues. The less predicted escape and more mutational robustness epitopes were prioritized in the final construction of multi-epitope vaccines [28].

### 2.14. Multi-Epitope Vaccine Construction

The prioritized B-cell (8 epitopes), CTL (8 epitopes), and HLT (6 epitopes) vaccine candidates were subsequently concatenated in sequence by introducing appropriate rigid and flexible linker peptides to create the final chimeric MULTI-EPITOPE vaccine construct. Human $\beta$-defensin-2 was attached to the N-terminus of the primary epitope chain with a rigid EAAAK linker to increase the immunogenicity and promote the recruitment of innate immune responses. In order to process efficiently and to reduce the immunogenicity of the junctions, B-cell epitopes were linked to each other by KK (lysine-lysine) linkers, CTL epitopes by AAY linkers, and HTL epitopes by GPGPG linkers. A 6xHis tag was added to the C-terminus to ease the purification process of the recombinant protein and its detection in experimental work [29].

### 2.15. Antigenicity and Allergenicity Assessment of the Final Vaccine

The complete multi-epitope vaccine construct, comprising an N-terminal human β-defensin-2 adjuvant, EAAAK, KK, AAY, and GPGPG linkers, and a C-terminal 6 × His tag, was evaluated for its overall antigenicity and allergenic potential. The antigenicity was predicted by VaxiJen v2.0 (https://www.ddg-pharmfac.net/vaxijen/VaxiJen/VaxiJen.html, Accessed on 26 July 2026), and the allergenicity was predicted by AllerTOP v2.1 (https://www.ddg-pharmfac.net/allertop_v2, Accessed on 26 July 2026). The analyses were carried out on the fully assembled construct to assess whether the incorporation of the adjuvant, linkers, and purification tag had any negative impact on the predicted immunological profile. The final construct was manually rechecked for correct epitope order, linker placement, unintended sequence duplication, and continuity of the coding sequence to ensure an uninterrupted reading frame [20,21].

### 2.16. Physicochemical Characterization

The final multi-epitope vaccine construct was fully characterized in terms of its physicochemical properties via the ExPASy ProtParam server (https://web.expasy.org/protparam, Accessed on 27 July 2026). Some important parameters used for evaluation were molecular weight, theoretical isoelectric point (pI), the total number of positive and negative residues, instability index, aliphatic index, grand average of hydropathicity (GRAVY), and estimated in vitro and in vivo half-life of the protein. For in vitro stability prediction, the instability index was used, with a value below 40 representing a stable protein, and for evaluation of the overall hydrophobicity, the aliphatic index was used, as well as the GRAVY score [30].

### 2.17. Secondary and Tertiary Structure Prediction

The structure of the final multi-epitope vaccine construct was predicted with the PSIPRED server (https://bioinf.cs.ucl.ac.uk/psipred, Accessed on 28 July 2026), and the secondary structural features were determined at the residue level. To overcome the three-dimensional architecture, a high-resolution atomic model of the chimeric construct was generated by AlphaFold (https://alphafold.ebi.ac.uk, Accessed on 28 July 2026), which showed the spatial folding, domain organization, and the relative orientation of the epitope arrays, junctional linkers, and the N-terminal adjuvant. The structural models were then analyzed to ensure the conformation of the B-cell, CTL, and HTL epitopes for best presentation to optimal immune receptors [31,32].

### 2.18. Structural Quality Assessment

The structural model of the multi-epitope vaccine construct was subjected to a thorough stereochemical quality control and refinement before molecular docking studies and receptor-interaction analysis. A Ramachandran plot was created by evaluating overall structural reliability and backbone dihedral angle distributions with PROCHECK (https://saves.mbi.ucla.edu, Accessed on 29 July 2026). The proportion of residues in the most favored, additionally allowed, generously allowed, and disallowed regions was quantified. Only validated models that exhibited the best geometric stability and high-confidence stereochemical profiles were chosen for further molecular docking and immune simulation study [33].

### 2.19. Molecular Docking with Receptor TLR7

Protein-protein molecular docking was used to examine the binding of the validated multi-epitope vaccine construct with the human innate immune receptor Toll-like receptor 7 (TLR7 (PDB ID: 7YTP). The crystal structure of human TLR7 was downloaded from the Protein Data Bank (PDB), dewatered and de-heteroatomized, and was energy-minimized using standard procedures. The optimized vaccine model was then docked to the TLR7 molecule with the help of the ClusPro 2.0 server (https://cluspro.org), which yielded multiple reasonable binding poses of the vaccine-TLR7 complex. Docking clusters were ranked by cluster population size, lowest energy score, and weighted electrostatic/van der Waals (WEDW). The highest-ranked complex with the most favorable structural characteristics, the best surface complementarity, and interacting surface residues was chosen for further molecular dynamics simulation and binding free energy calculations. Among the generated docking solutions, Cluster 11 was selected as the lowest-energy representative complex and was used as the starting structure for the subsequent 100 ns molecular dynamics simulation [34].

### 2.20. Molecular Dynamics Simulation

The structural stability and dynamic behavior of the vaccine–TLR7 complex were studied by carrying out a 100 ns molecular dynamics (MD) simulation in the Desmond module of the Schrödinger suite (https://www.schrodinger.com/platform/products/desmond, Accessed on 30 July 2026). The complex was packed into an orthorhombic box for simulation with a buffer of 10 Å filled with the TIP3P water model with corresponding counter-ions to neutralize the system. To reach physiological ionic strength, NaCl was used to yield a final concentration of 0.15 M. The system was energy-minimized and equilibrated with the Desmond standard relaxation protocol before being simulated. The production run of 100 ns was carried out at 300 K and 1.0 bar, with the Nose–Hoover chain thermostat and Martyna–Tobias–Klein barostat controlling the temperature and pressure, respectively. The long-range electrostatic interactions were calculated with the Ewald method and a cutoff distance of 9.0 Å. The root mean square deviation (RMSD), root mean square fluctuation (RMSF), radius of gyration (Rg), intermolecular hydrogen-bond dynamics, and energy fluctuations were included in trajectory analysis. The following parameters were used to describe the conformational stability, flexibility at the residue level, structural compactness, and persistence of interactions between the vaccine and TLR7 throughout the simulation [35].

### 2.21. Immune Simulations

Immune response to the designed multi-epitope vaccine was simulated using the C-ImmSim online server (https://kraken.iac.rm.cnr.it/C-IMMSIM, Accessed on 1 August 2026), which is a model of cellular and humoral immune response. Immune-response profiles were generated from the simulation, and the length of time simulated was about 12 weeks (84 days). Vaccine-induced changes in B-cell, helper and cytotoxic T-cell, NK-cell, macrophage and dendritic-cell populations were monitored, together with immunoglobulin production, antigen clearance, and cytokine responses throughout the simulation period [36].

### 2.22. Software and Computational Resources

MAFFT (FFT-NS-2), EMBOSS Cons, EMBOSS vaxiJen v2.0, AllerTOP v2.1, IFNepitope, IL4pred, IL10pred, ClusPro 2.0, Desmond (Schrödinger) and C-ImmSim were used in the computational analysis. Sequences and structures were retrieved from NCBI GenBank and PDB, respectively. Versions of software are reported, if available, and web servers are reported in the versions available at the time of analysis, which are updated regularly.

## 3. Results

### 3.1. Sequence Retrieval and Core Dataset Curation

120 sequences of the Betacoronavirus spike protein were downloaded to create an initial sequence set for comparison. This dataset contained multiple coronavirus groups such as SARS-CoV-2, human coronaviruses OC43 and HKU1, Bovina virus, Pangolin virus, and an unclassified Betacoronavirus sequence. The largest proportion of data was SARS-CoV-2 (100 sequences), with other sequences representing other betacoronaviruses from humans and animals. The lengths of the sequences spanned from 1241 to 1363 amino acids, with an average length of ~1279 amino acids, which is in accordance with the expected size range of coronavirus spike glycoproteins. Two of them were annotated as partial sequences. Five more records were not annotated at the species level in the FASTA header and contained three PDB-derived spike chains and two patent-associated sequences. Multiple-sequence alignment and comparative conservation analysis were then carried out using the curated sequence collection as the input dataset. Table 1 below represents the composition of retrieved sequences of spike proteins.

### 3.2. Multiple Sequence Alignment (MSA) and Structural Superposition

The 120 spike protein sequences were aligned using multiple sequence alignment (MSA), yielding a length of 1501 positions; each ungapped protein had a sequence length ranging from 1241 to 1363 amino acids. Although there was some variation in the sequences, there was considerable conservation in the alignment, with about 90.7% average conservation per residue. There were 790 positions that were conserved at ≥90% with 538 positions conserved at ≥95%. Similar conserved sequence regions were also observed in all 120 proteins, with sequence lengths obtained by conservation-based extraction (extracts) ranging from 1023 to 1063 amino acids. These results were used to pinpoint an extensive conserved sequence backbone for further Prioritization of functionally constrained spike regions. The summary of the multiple sequence alignment is given in Table 2 below.

**Figure 1:**
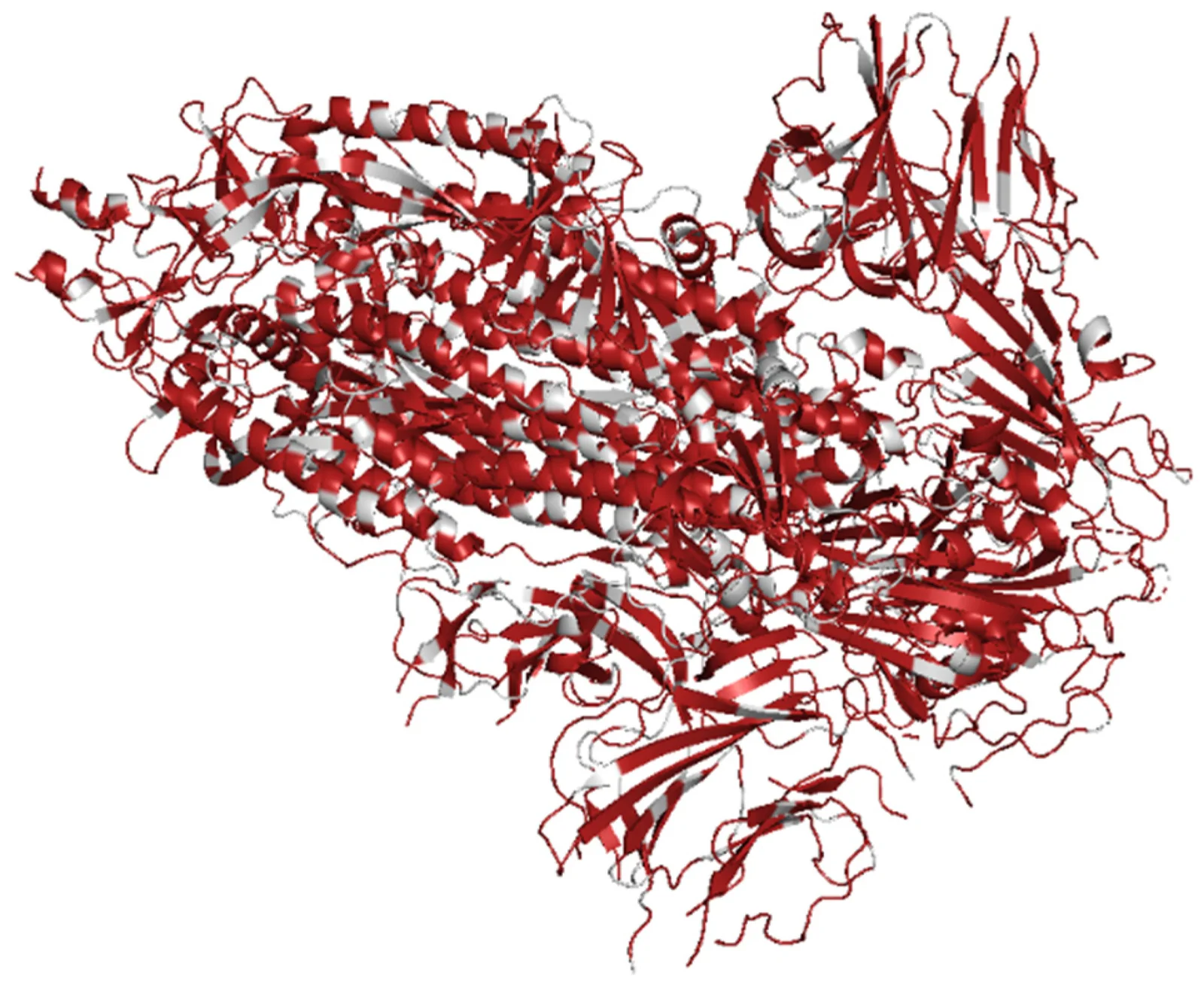
Structural superposition of representative Betacoronavirus spike proteins showing conservation of the overall structural framework. Superimposed structures are displayed in contrasting colors to visualize structurally conserved regions and localized conformational variation.

Structural superposition was then used to determine if there was any conservation of the overall spike protein structure. Superimposed structures indicated a significant degree of commonality in the basic structure, but there were localized differences in those areas that are structurally less conserved (Figure 1). The sequence and structure analysis was then used to identify conserved, structurally constrained regions that served as a basis for further identification of conserved regions in the coronavirus fusion machinery.

**Figure 2:**
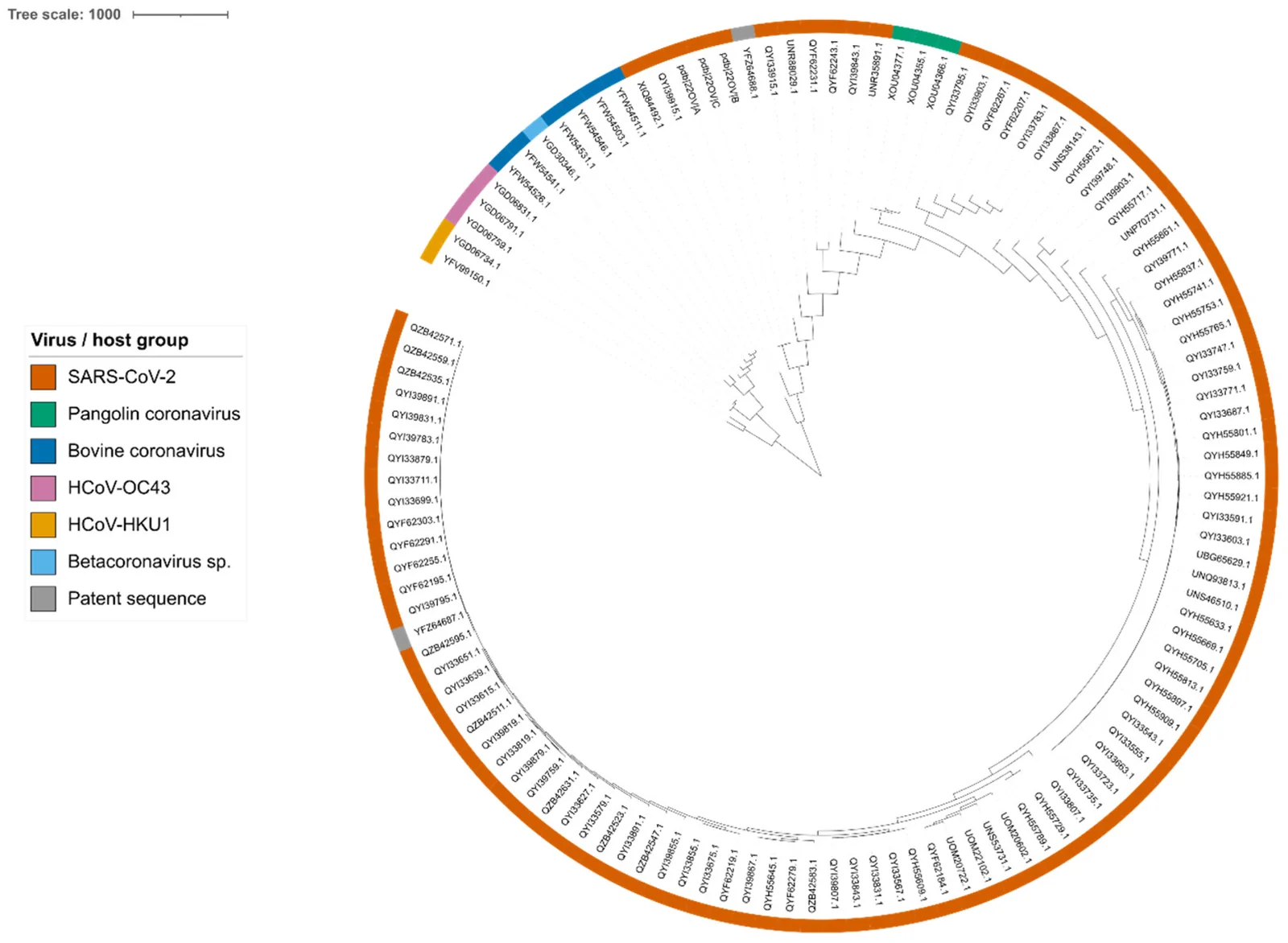
Phylogenetic relationships among the 120 Betacoronavirus spike protein sequences. Branch-tip colors indicate the corresponding virus or host-associated group, including SARS-CoV-2, pangolin coronavirus, bovine coronavirus, HCoV-OC43, HCoV-HKU1, Betacoronavirus sp., and sequences lacking species-level annotation. The scale bar represents evolutionary distance.

The spike protein sequences showed clear clustering and groups, corresponding to the coronavirus group represented (Figure 2). The SARS-CoV-2 sequences comprised the major and most similar cluster; the sequences were highly similar in this dataset. Pangolin coronavirus, bovine coronavirus, HCoV-OC43, HCoV-HKU1, and other sequences from the Betacoronavirus family were clustered in more distant branches, suggesting higher evolutionary distance from the cluster dominated by SARS-CoV-2. Bovine coronavirus and the embecoviruses OC43 and HKU1 of humans were found to cluster in different lineages of the tree, and sequences of pangolin coronavirus were located within the expanded Sarbecoviruses-associated portion of the tree. Phylogenetic topology showed overall good conservation and significant sequence differences between all of the Betacoronavirus groups represented, which drove the search for conserved regions that were retained through evolutionary distinct spike proteins.

### 3.3. Pan-Betacoronavirus Conservation and Entropy Profiling

**Figure 3:**
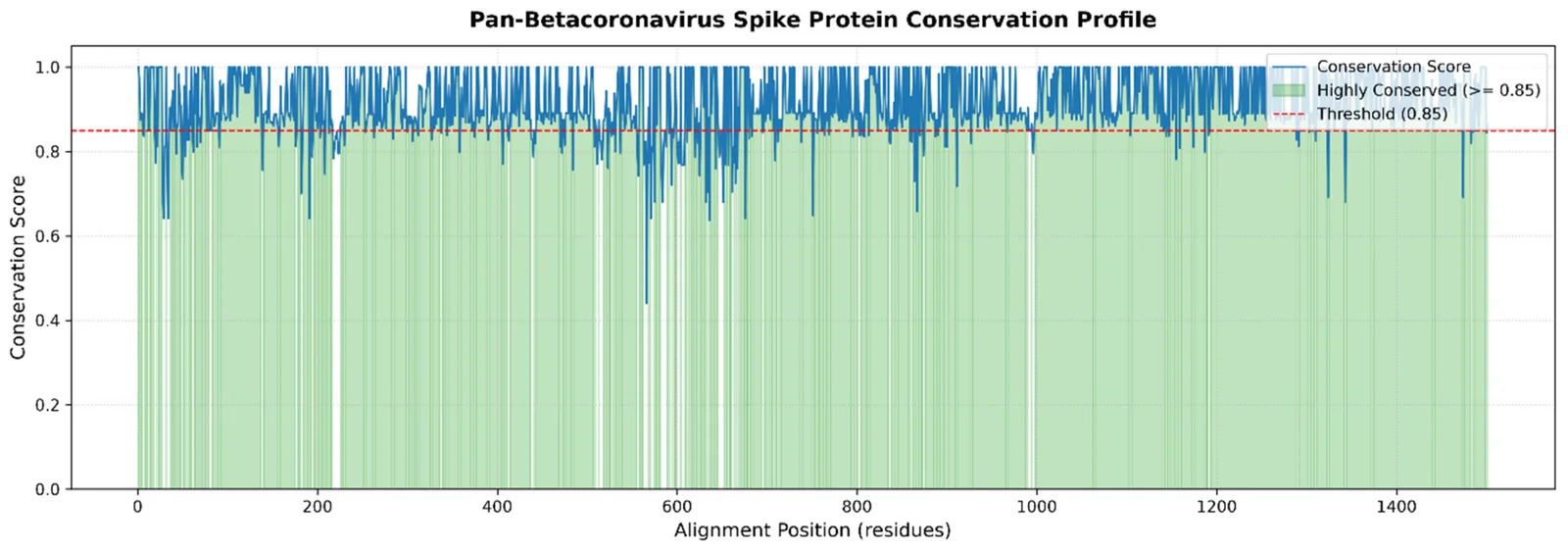
Residue-wise conservation profile of the Betacoronavirus spike protein multiple sequence alignment. The blue line represents the conservation score at each alignment position, while green shading indicates highly conserved residues with scores ≥ 0.85. The red dashed line denotes the conservation threshold of 0.85.

Conservation analysis of the 1501-position multiple sequence alignment by residue showed that there is a high degree of conservation across the spike protein dataset (Figure 3). The mean conservation score was 0.915, with all scores from 0.440 to 1.000. With a conservation threshold of ≥0.85, the number of highly conserved positions was 1287 (85.7%). Moreover, 666 positions (44.4%) showed conservation scores ≥0.90, 587 positions (39.1%) exceeded 0.95, and 461 positions (30.7%) were completely conserved with a score of 1.00. A number of long segments of high conservation were observed along the alignment, one of which was a continuous stretch of 96 residues at positions 1193–1288, and others at positions 998–1063 and 1374–1440. The summary of conservation is given in Table 3 below.

However, localized decreases in conservation were seen at discrete sites, suggesting areas of increased sequence variability. The sequence-level basis for subsequent identification and prioritization of structurally constrained epitopes of the spike fusion machinery was given by these conserved segments.

### 3.4. Evolutionary Pressure and Recombination Screen

**Figure 4:**
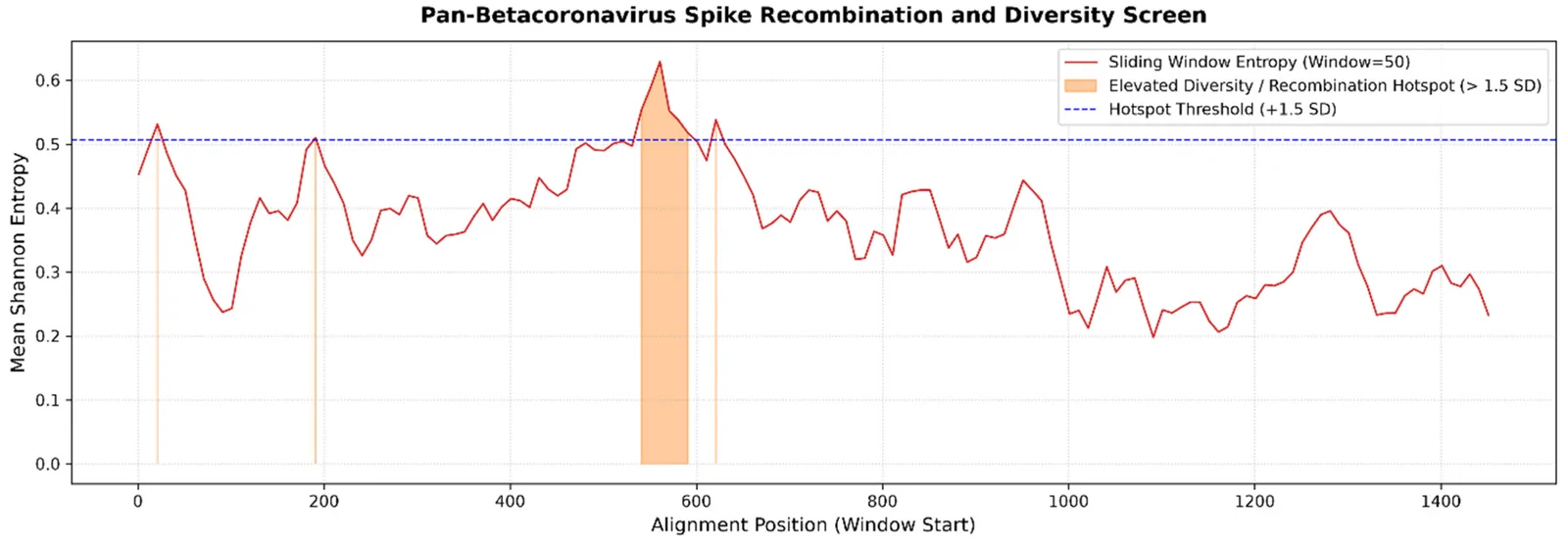
Sliding-window analysis of spike sequence diversity across pan-Betacoronavirus alignment. Mean Shannon entropy was calculated using 50-residue sliding windows. The dashed line indicates the +1.5 SD diversity threshold (0.507), while shaded regions represent windows with elevated sequence diversity and potential evolutionary/recombination hotspots.

To identify regions with higher sequence diversity that may represent evolutionary or recombination-prone variability, sliding window Shannon entropy was calculated across the aligned spike sequences. Across 146 overlapping 50-residue windows, the mean entropy was 0.369 ± 0.092, with values ranging from 0.198 to 0.629. Nine windows had increased diversity based on the threshold of 1.5 standard deviations above the global mean (0.507). The strongest and most consistent signal of diversity was found in a window of 129 positions spanning from 541 to 670, while the other windows were grouped into three regions between 21 and 70, 191 and 240, and 541 and 670 positions. The Maximum entropy was found at position 561 with a window length of 41, at 0.629. Generally, the entropy of the C-terminal end of the alignment was lower, representing relatively strong sequence stability. The results reveal localized areas of evolutionary hotspots as well as the presence of more widespread conserved regions for which downstream conserved-epitope prioritization can be made. Of course, high entropy does not necessarily mean recombination, but rather it is indicative of a diversity-based recombination screen, so these areas were considered as potential evolutionary hotspots for further analysis. Sliding-window analysis of spike sequence diversity across pan-Betacoronavirus is given in the Figure 4.

### 3.5. Antigenicity, Allergenicity, and Toxicity Profiling

The conserved spike protein sequence was assessed for antigenicity, allergenicity, and toxicity for further epitope screening. The overall antigenicity score of the 1063-amino-acid conserved protein was 0.4628, which is higher than the viral antigen cut-off value of 0.4 and was classified as a probable viral antigen by VaxiJen v2.0. The sequence was predicted as a probable non-allergen by allergenicity assessment by AllerTOP v2.1. Toxicity profiling was also performed, confirming the non-toxicity of the conserved protein. The sequence analyzed in the study had a predicted molecular weight of around 117.89 kDa, net charge of −12.13, and hydrophobicity value of 0.04. Together, these predictions suggested that the conserved sequence derived from the spike had antigenic potential, but no predicted allergenic or toxic properties, and thus is suitable for subsequent identification and prioritization of conserved immune epitopes.

### 3.6. B-Cell Epitope Prediction and Screening

**Figure 5:**
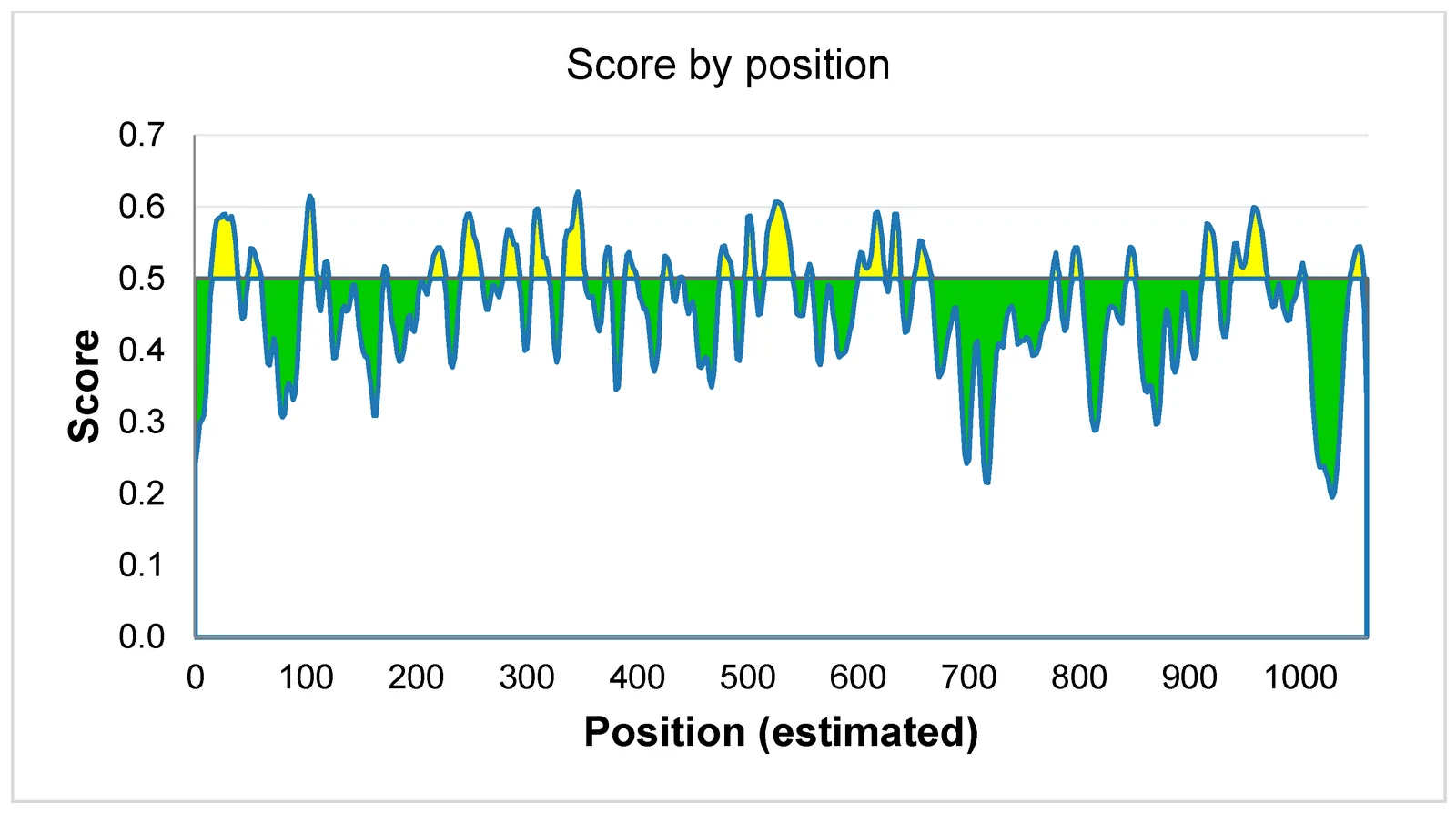
Residue-wise linear B-cell epitope prediction profile of the conserved Betacoronavirus spike-derived sequence. The blue line represents the prediction score across individual residue positions, while regions exceeding the 0.50 prediction threshold indicate candidate B-cell epitope-associated regions selected for subsequent screening.

Linear B-cell epitope prediction of the conserved spike-derived sequence identified 32 candidate regions, including peptides of 1–33 amino acids in length. The residue-wise prediction profile was 0.459, with a range between 0.183 and 0.630. Those candidate regions that surpassed the threshold for prediction were then screened rigorously for antigenicity, allergenicity, toxicity, and sequence conservation. After this filtering, we have 8 B-cell epitope candidates that are probable antigens, probable non-allergens, and non-toxic peptides that show 95.5% sequence conservation. They had VaxiJen scores ranging from 0.556 to 0.668, with IWLGFIAGLI being the most antigenic (0.668), followed by SFVIGEVQIAPGQT (0.594) and GTNTSQVAVLYQV (0.577). Residue-wise linear B-cell epitope prediction is given in the Figure 5.

The retained epitopes were spread throughout the conserved spike sequence and were good candidates for subsequent immunization and structural prioritization. Prioritized B-cell epitopes are given in Table 4 below.

### 3.7. CTL Epitope Prediction and Screening

The conserved sequence from the Spike was screened for 10 high-affinity peptide–HLA class I combinations by CTL epitope screening. The predicted binding affinities were between 0.939 and 10.315 nM, reflecting strong binding of the peptides to the associated HLA-I alleles. Nine safe peptide–HLA combinations (corresponding to eight unique CTL epitopes) were identified in subsequent screening for antigenicity, allergenicity and conservancy. The retained epitopes were all predicted to be antigenic, non-allergenic, and non-toxic and completely conserved within the sequence set analyzed. Of these, FQFCDPFLV had the highest predicted binding affinity to HLA-A*02:06 (0.939 nM) and was also predicted to bind to HLA-A*02:01 (5.599 nM). Other high-affinity candidates included EVFNAFASV–HLA-A*68:02 (2.026 nM), SQVAVLYQV–HLA-A*02:06 (3.577 nM), and CPFEVFNAF–HLA-B*35:01 (3.695 nM). The candidate that was excluded due to the predicted toxicity was MTSCCSCLK, which had good antigenicity and HLA binding. The screening steps resulted in a narrowed-down list of conserved CTL epitopes for further HLA coverage and vaccine construct prioritization. Prioritized CTL epitopes are given in Table 5 below.

### 3.8. HTL Epitope Prediction and Screening

The conserved spike-derived sequence was screened for helper T-lymphocytes (HTLs) using epitope screening, and 10 peptide–HLA class II combinations were identified. Predicted binding values ranged from 28 to 340 nM, with DLPIGIITRFQLLAL–HLA-DPA102:01/DPB101:01 having a predicted binding of 46 nM and DLPIGIITRFQLLAL–HLA-DPA102:01/DPB101:01 having a predicted binding of 28 nM being the highest predicted binding. After screening for antigenicity, allergenicity, toxicity, and conservancy, nine peptide–HLA II combinations were safe and represent six different HTL epitopes. The expected antigenic, non-allergenic, non-toxic, and conserved nature of all selected epitopes was 100% in all the analyzed data. The predicted HLA-II binding was broadest for the peptide DLPIGIITRFQLLAL, which binds three different HLA-II molecules, and KNDGYFKIYSKHTPI was predicted to bind two HLA-DRB1 alleles. Two candidates were removed for predicted toxicity (ECSNLLLQYGSFCTQ) and/or for lack of antigenicity, HLA-II binding, and conservancy (NAALKWVHRL). These results led to a small list of conserved HTL candidates which were then prioritized for immune-response and vaccine construct development. The identified prioritized HTL epitopes are given in Table 6 below.

### 3.9. Population Coverage Analysis

**Figure 6:**
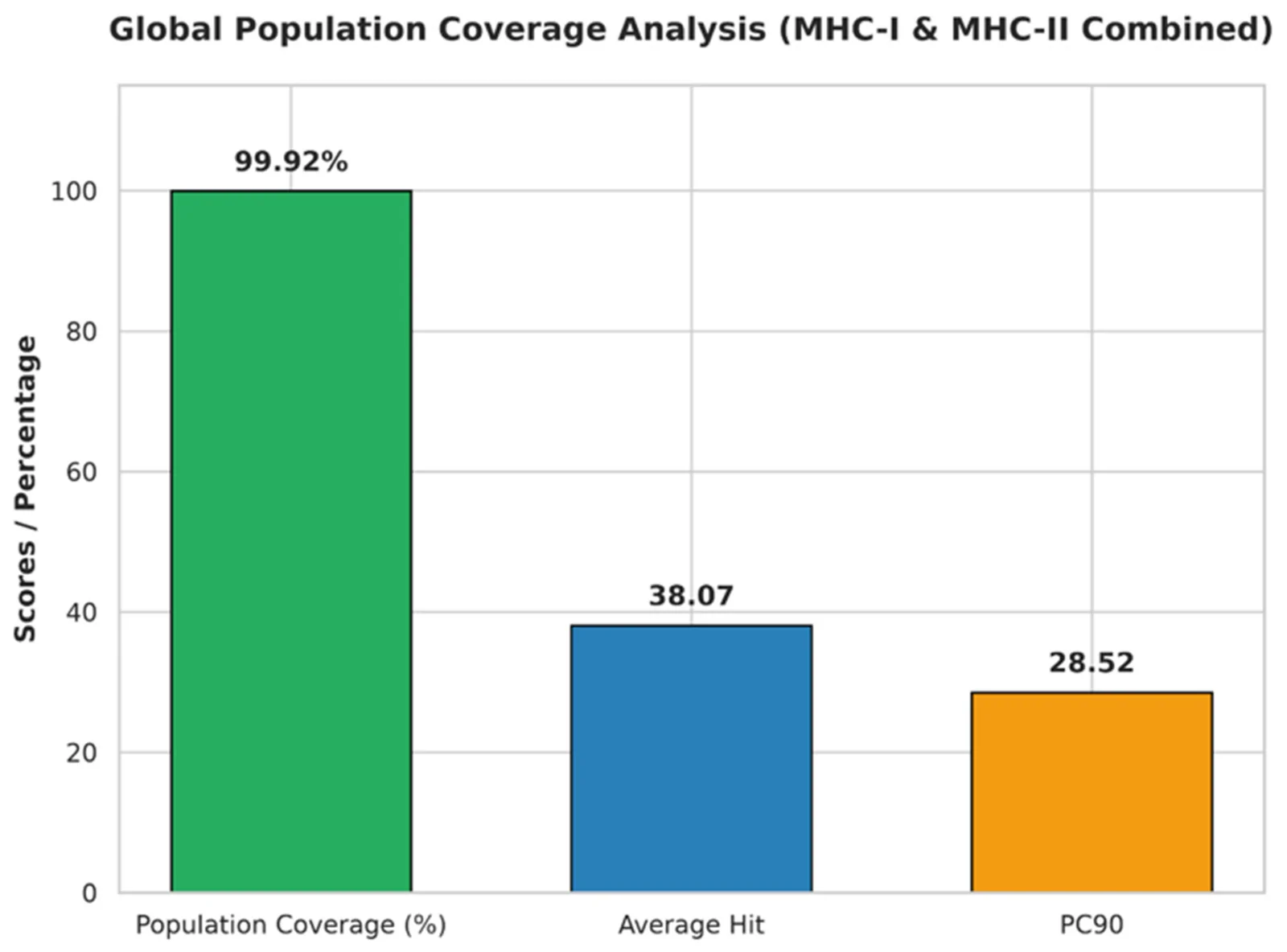
Predicted global population coverage of the selected MHC-I- and MHC-II-restricted epitopes.

Following immunogenic screening, the prioritized CTL and HTL epitopes were evaluated for their predicted population coverage based on the associated MHC class I and class II alleles. Combined MHC-I and MHC-II analysis using the IEDB Population Coverage tool predicted a global population coverage of 99.92%. The average number of epitopes–HLA combinations recognized per individual was estimated at 38.07, while the PC90 value was 28.52, indicating that 90% of the global population was predicted to recognize at least approximately 28 epitope–HLA combinations. These results represent theoretical HLA population coverage derived from allele-frequency-based computational analysis and should not be interpreted as evidence of population-level immunogenicity or protection. Figure 6 below represents the worldclass population coverage, average hit and PC90 computed by IEDB.

### 3.10. Mutational Scanning and Escape Vulnerability Analysis

**Figure 7:**
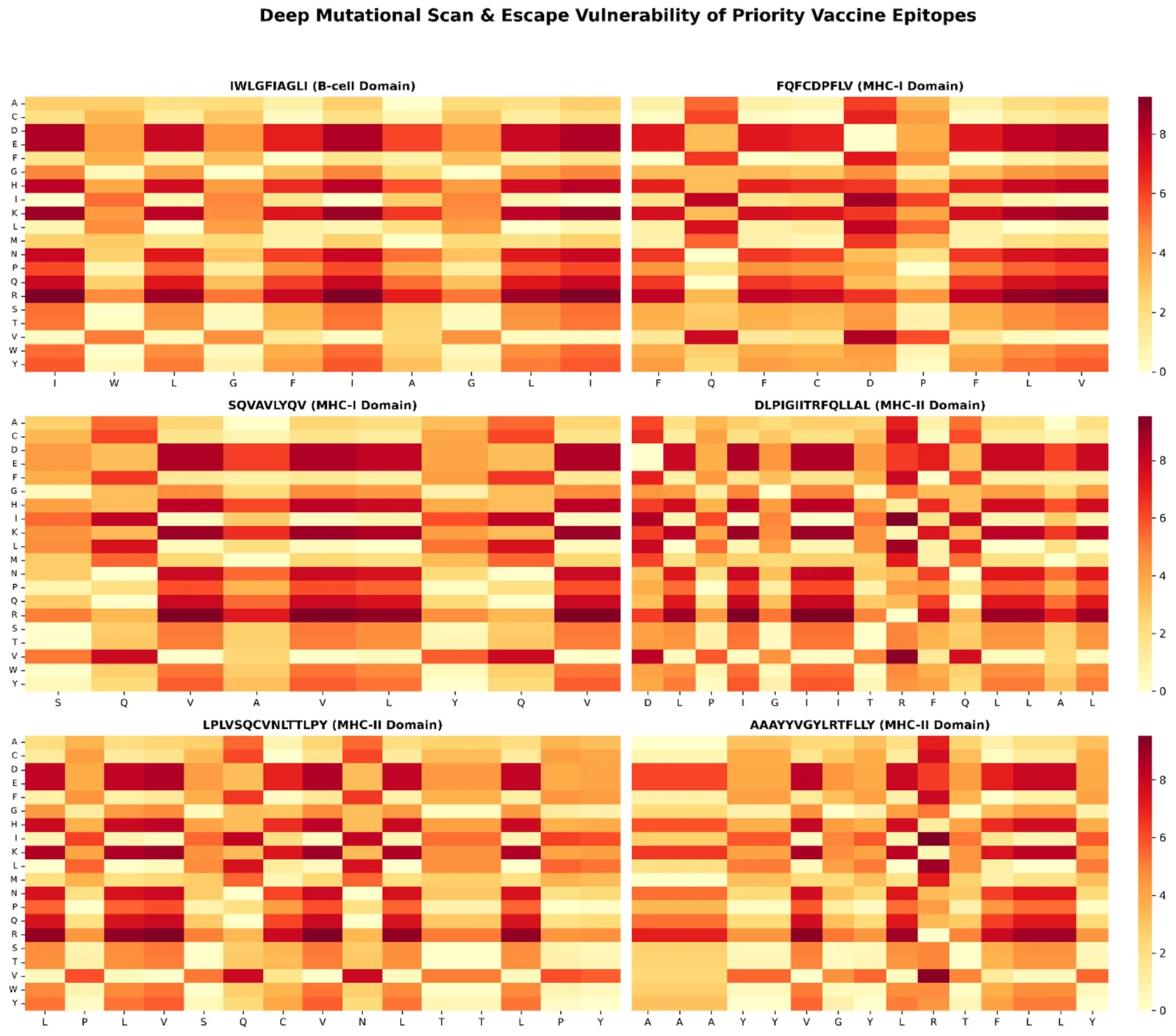
Deep mutational scanning and predicted escape vulnerability landscape of prioritized B-cell, MHC-I, and MHC-II vaccine epitopes. Each heatmap represents the effect of amino acid substitutions at individual epitope positions, with increasing color intensity corresponding to higher predicted escape vulnerability scores. The heterogeneous substitution patterns identify mutation-sensitive hotspots as well as comparatively mutation-tolerant positions within the prioritized epitopes.

Prioritized vaccine epitopes were scanned for individual amino acid changes using deep mutational scanning. The mutational library consisted of 540 substitution profiles over the analyzed epitopes. The vulnerability scores for escape mutations differed significantly for different residues on the backbone of the protein (as well as for different substituted amino acids at the same position), suggesting that the effect of a mutation was not evenly distributed across individual epitopes (Figure 7). The heatmap showed some hotspots, positions with mutations that created high escape vulnerability scores (>8), and others that were more tolerant of amino acid replacements.

Out of the CTL epitopes represented in the mutational library, the mean escape vulnerability score for FQFCDPFLV was 4.29, and for KIYSKHTPI and AAYYVGYLR were 4.26 and 3.87, respectively, without taking into account substitutions found in the wild type. Selected substitutions in KIYSKHTPI and AAYYVGYLR had the highest individual vulnerability scores of 9.54 and 9.26, respectively. Even with these substitution-sensitive areas, there was significant variation in the sensitivity of the amino acid substitutions, suggesting that not all changes in amino acids are expected to have the same effect. These findings allowed the identification of potential escape-sensitive residues and provided another criterion for prioritizing epitopes for the Preemptive vaccine construct, in terms of robustness in evolution.

### 3.11. Multi-Epitope Vaccine Construct Design

**Figure 8:**
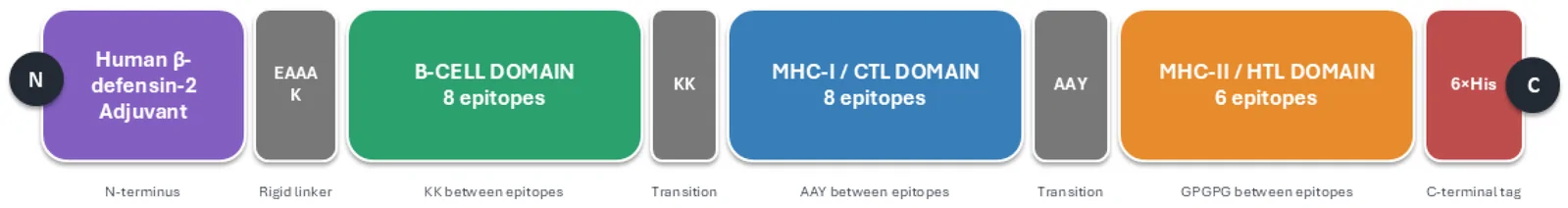
Vaccine design architecture representing the fusion of prioritized epitopes with linkers, adjuvant, and 6× His Tag.

After immunological screening and mutational evaluation, the prioritized epitopes were joined together into a multi-epitope vaccine construct that contains 8 B-cell, 8 CTL, and 6 HTL epitopes. Human β-defensin-2 was attached to the N-end of the epitope region as an immunostimulatory adjuvant with a rigid EAAAK linker. The B-cell epitopes formed a connected network via KK linkers, while the CTL epitopes were connected by AAY linkers for the purpose of separating the epitopes and proteasomal processing. The HTL epitopes were fused with GPGPG linkers to enable the presentation of the helper T-cell determinants to be carried out independently. The construct was sequentially designed in order to include adjuvant, B cell, MHC-I, and MHC-II domains in a single vaccine candidate that will contain predicted humoral and cellular immune determinants. A C-terminal 6× histidine tag was added for potential recombinant purification and detection. The final architecture would be a composite of conserved and screened epitopes and linkers specific to the function, for further physicochemical, structural, and immunological characterization. The vaccine design architecture is represented below in Figure 8.

### 3.12. Antigenicity and Allergenicity Profiling of the Vaccine Construct

The antigenic and allergenic potential of the designed multi-epitope vaccine construct was evaluated. The virus model (0.40) was used with VaxiJen v2.0 to get the final antigen construct as a probable antigen with an overall antigenicity score of 0.5215. This score surpassed the set threshold value for antigenicity, suggesting retention of predicted antigenicity after adding the β-defensin-2 adjuvant, epitope assembly, and incorporation of linkers. The full construct was also assessed for allergenicity using AllerTOP v2.1, a result that was consistent with the other assessments. The combined results showed that the formulated vaccine was expected to have good antigenicity and was not expected to cause allergic reactions, thus progressing it to further physicochemical and structural analysis.

### 3.13. Cytokine Induction Profiling

The cytokine-inducing potential of the prioritized epitopes was evaluated for IFN-γ, IL-4, and IL-10 responses. Of the 22 analysed epitopes, 19 (86.4%) were predicted as IFN-γ inducers, demonstrating a predominant IFN-γ-associated response across the selected epitope panel. All eight B-cell epitopes and all six MHC-II epitopes were predicted to induce IFN-γ, whereas five of the eight MHC-I epitopes were IFN-γ positive. The strongest IFN-γ predictions were observed for the MHC-II epitopes DLPIGIITRFQLLAL and LPLVSQCVNLTTLPY, each with a score of 0.977, followed by KNDGYFKIYSKHTPI (0.870).

IL-4 induction was predicted for 15 of 22 epitopes (68.2%), including four B-cell, six MHC-I, and five MHC-II candidates. AAAYYVGYLRTFLLY exhibited the highest IL-4 score (0.780), while FQFCDPFLV and AAYYVGYLR each showed scores of 0.700. In contrast, IL-10 induction was comparatively limited, with EVFNAFASV representing the only epitope classified as IL-10 positive, with a score of 0.561. Overall, the cytokine prediction profile was dominated by IFN-γ-inducing epitopes, with additional IL-4-associated responses across multiple B-cell and T-cell candidates. The cytokine-induction profiles of the selected epitopes are given in Table 7 below.

### 3.14. Physicochemical Profiling of the Designed Vaccine Construct

ExPASy ProtParam was used to evaluate physicochemical properties of the final multi-epitope vaccine construct. The construct contained 409 amino acids, a predicted molecular weight of 45.13 kDa, and a theoretical isoelectric point (pI) of 9.39, showing an overall basic nature of the construct. There were a total of 42 positively charged residues (Arg, Lys) and 18 negatively charged residues (Asp, Glu) in the sequence. The instability index predicted was 27.16, which was considered a stable construct. The aliphatic index (72.79) shows that the protein had a significant aliphatic side chain contribution, and the overall hydrophobicity of the protein could be described by the GRAVY value of −0.052, which represents a close-to-even balance. In mammalian reticulocytes in vitro, the half-life was estimated to be 30 h and in yeast in vivo >20 h, while in Escherichia coli in vivo >10 h. These in silico predicted physicochemical properties together pointed to a stable vaccine construct appropriate for progression to the structural modeling and in silico characterization. Predicted physicochemical properties of the multi-epitope vaccine construct are given in Table 8 below.

### 3.15. Structural Modelling and Conformational Screening

**Figure 9:**
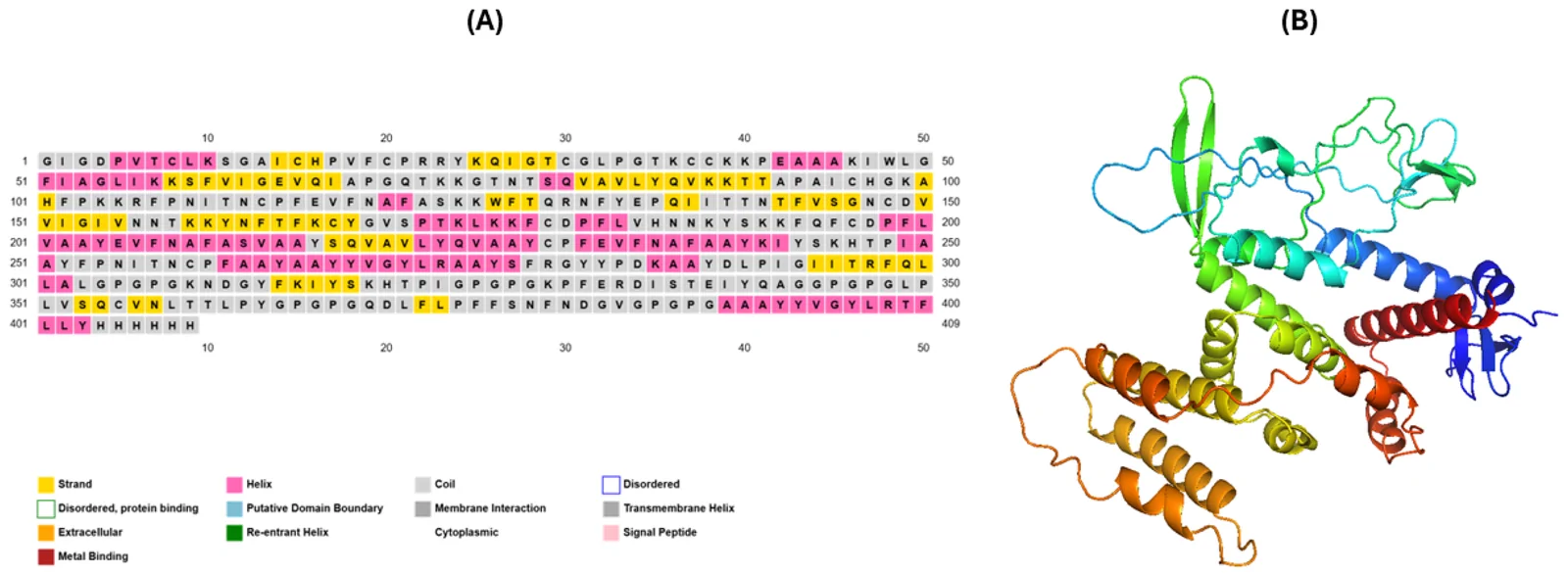
Structural modeling of the designed multi-epitope vaccine construct. (**A**) Residue-level secondary-structure prediction showing the distribution of α-helices, β-strands, coils, and disordered regions across the vaccine sequence. (**B**) Predicted three-dimensional structure of the vaccine construct displayed in cartoon representation, illustrating its overall fold and distribution of helical, β-sheet, and loop regions.

The designed multi-epitope vaccine construct was subjected to secondary- and tertiary-structure analyses to determine its conformational organization. The secondary-structure profile showed that the 409-amino-acid sequence was heterogeneous, with the presence of several regions of α-helicity, β-strand, coil, and disordered regions (Figure 9A). Helical segments populated the construct, and multiple β-strand regions with intervening coil/disordered segments contributed to structural diversity. The regions predicted for the presence of linkers also added flexible domains between the different epitope domains, which was in line with the modular structure of the vaccine construct. Three-dimensional structural modeling resulted in the folded structure with specific α-helices, β-sheets, loops, and extended coil areas (Figure 9B). The predicted model showed a small central architecture surrounded by several flexible protrusions that extended outward, which was in line with the heterogeneous secondary-structure composition of the sequence of MEs. In general, the structural prediction showed that the assembly of the adjuvant, linkers, and selected peptides was feasible to create an organized 3D conformation. The predicted model was then structurally analyzed for quality and refined prior to receptor binding analysis.

### 3.16. Structure Quality Assessment

**Figure 10:**
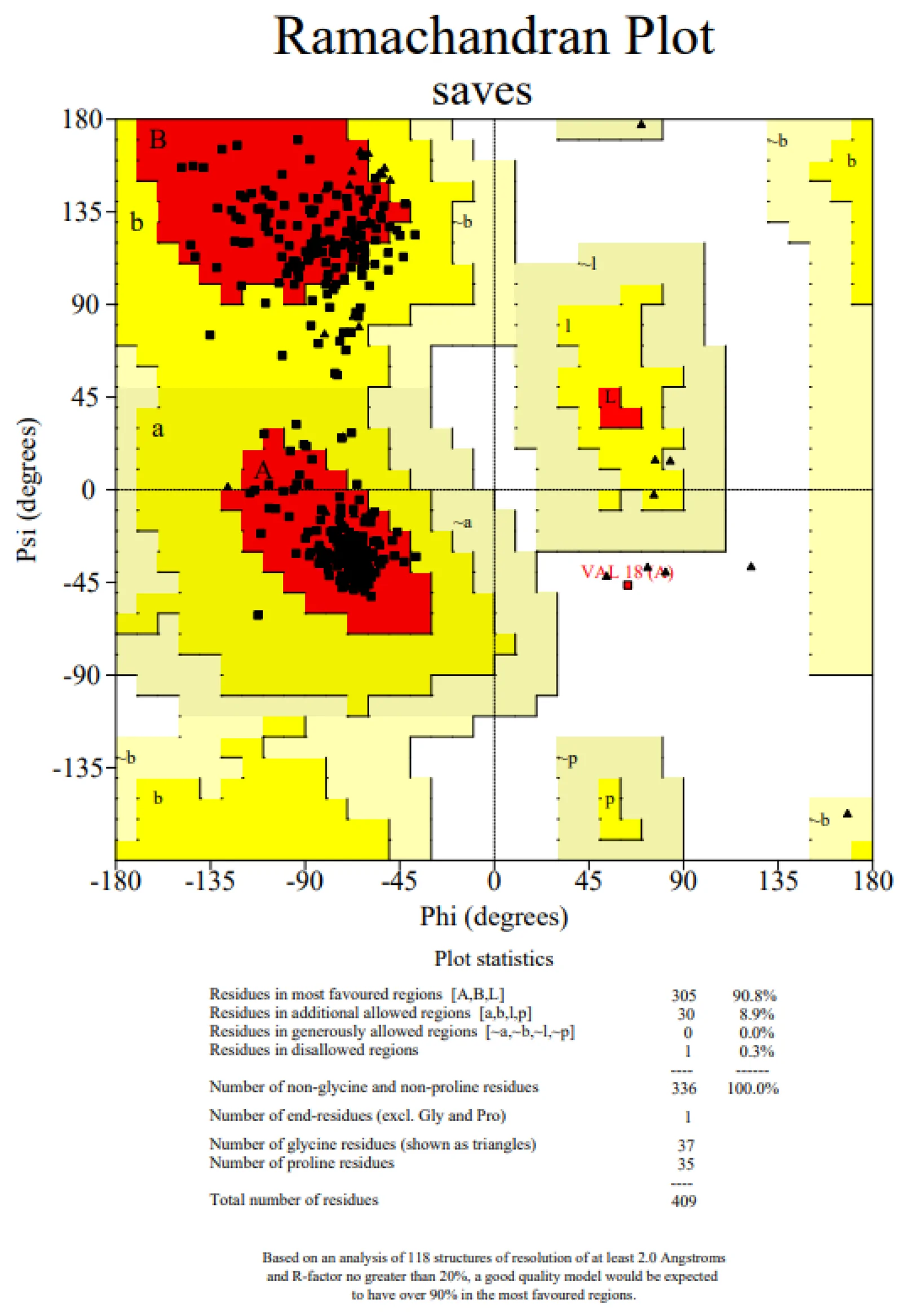
Ramachandran plot-based stereochemical validation of the predicted multi-epitope vaccine structure using PROCHECK. The analysis placed 90.8% of residues in the most favored regions and 8.9% in additionally allowed regions, while only 0.3% occurred in a disallowed region, indicating an overall acceptable stereochemical geometry of the predicted model.

The structure of the predicted vaccine was analyzed for stereochemical quality using the PROCHECK Ramachandran plot. The 336 non-glycine and non-proline residues were all within the most favored regions (305, 90.8%), with 30 residues (8.9%) within additionally allowed regions. There was no residue found in the most generous areas and only one residue (0.3%) in a disallowed area. So, 99.7% of the inspected residues were located in preferred or extra permitted conformational areas. The majority of residues were in the stereochemically acceptable regions, and a relatively small percentage of residues were found outside the Ramachandran regions, which warranted the overall geometric quality of the predicted 409-residue vaccine model for further receptor-interaction and molecular simulation studies. The constructed Ramachandran Plot is given in Figure 10 below.

### 3.17. Molecular Docking with Human TLR7

**Figure 11:**
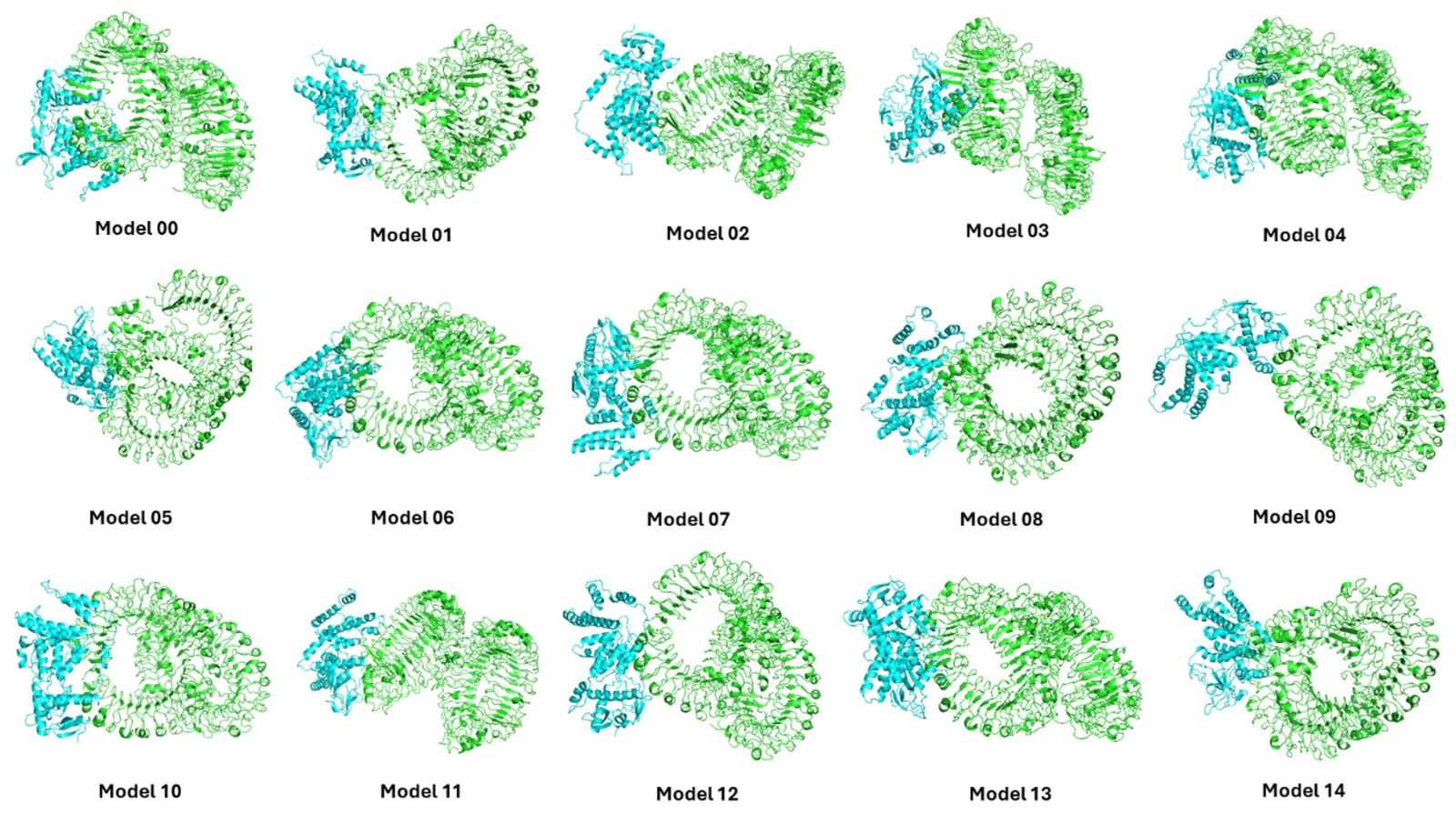
Predicted docking conformations of the multi-epitope vaccine construct with human TLR7 generated using ClusPro. Models 00–14 illustrate representative docking orientations of the vaccine construct (cyan) relative to TLR7 (green), demonstrating alternative predicted receptor-binding configurations.

To study the predicted interaction of the designed multi-epitope vaccine construct with human Toll-like receptor 7 (TLR7), protein–protein docking was conducted. Multiple candidate binding orientations were generated by ClusPro with different relative orientations of the vaccine construct at the surface of TLR7 (Figure 11). A total of 30 docking clusters were retrieved, including clusters of varying population (11–45 members). The largest (Cluster 0) had 45 members and had a representative weighted score of −1081.9. There were 45 members in cluster 1 with a representative score of −1043.3.

Several other docking orientations had better ClusPro scores. Cluster 11 produced the lowest-energy pose among the reported solutions, with a score of −1219.1, followed by Cluster 6 (−1218.0), Cluster 5 (−1204.3), and Cluster 10 (−1194.2). Multiple high-ranked docking orientations suggested several docking modes between the vaccine construct and TLR7 that were computationally favorable. The analysis of these structures collectively indicated good structural compatibility between the vaccine construct and TLR7 and provided potential complexes for further docking and stability analysis.

### 3.18. Molecular Dynamics Simulations

**Figure 12:**
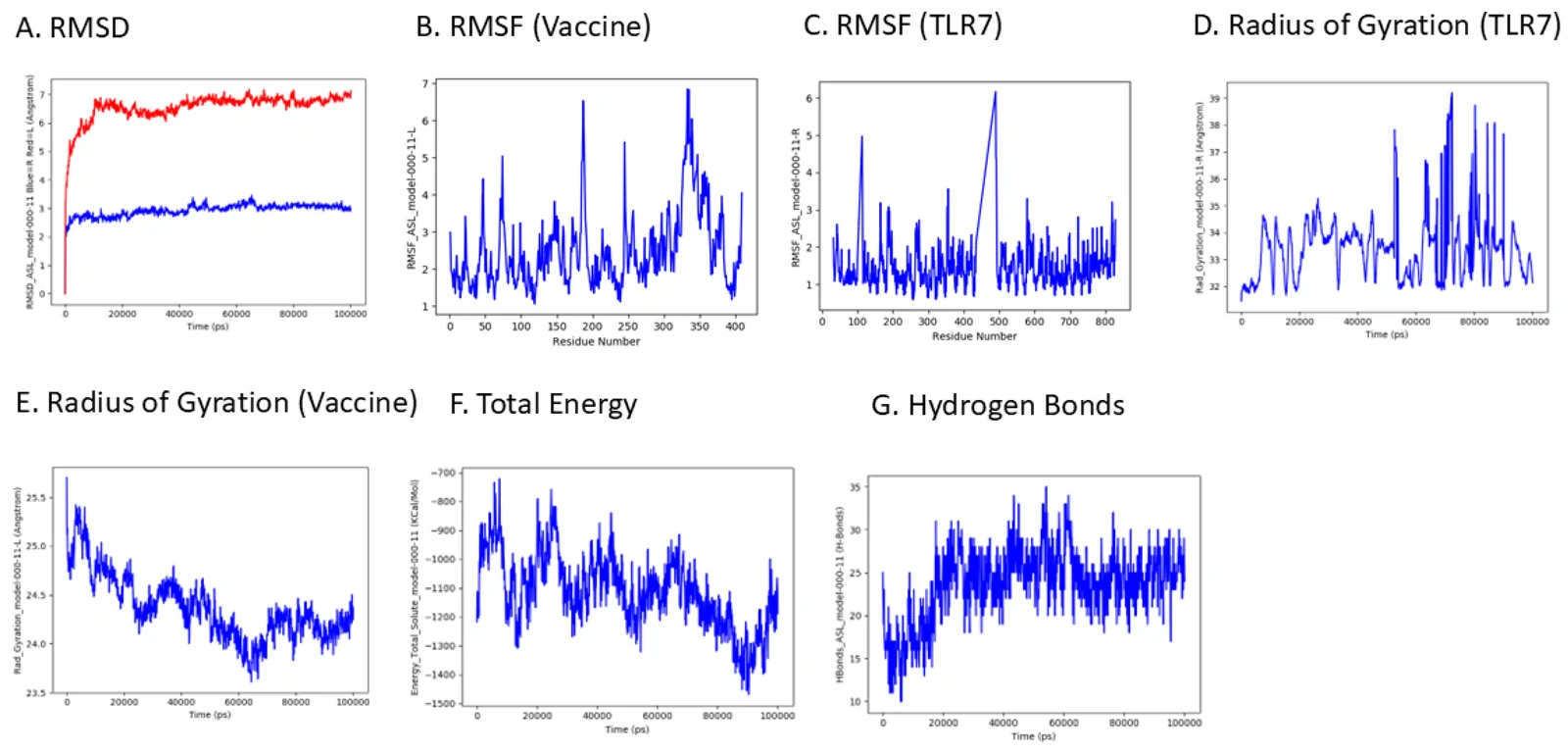
Molecular dynamics analysis of the vaccine–TLR7 complex over 100 ns. (**A**) RMSD trajectories of the vaccine construct and TLR7. (**B**,**C**) Residue-wise RMSF profiles of the vaccine and TLR7, respectively. (**D**,**E**) Radius of gyration profiles describing conformational compactness of TLR7 and the vaccine construct. (**F**) Total solute energy throughout the simulation. (**G**) Hydrogen-bond profile of the vaccine–TLR7 complex during the 100 ns trajectory.

The dynamic behavior and stability of the vaccine–TLR7 complex were evaluated through a 100 ns molecular dynamics simulation. Using RMSD analysis, the initial structural adjustment was found to be followed by stabilization of both interacting components (Figure 12A). The mean RMSD of the TLR7 component was 2.93 Å, with a range of 3.46 Å after equilibration, a relatively narrow range. The trajectory of the vaccine construct was more conformational, with an average RMSD of 6.55 Å and a maximum RMSD of 7.18 Å, and was comparatively more stable in the later part of the simulation. Median level RMSF (Figure 12B,C) showed mostly moderate fluctuations with some flexible regions. The RMSF values were about 2.06 Å for the vaccine construct and 1.33 Å for TLR7, with peak values of 7.39 Å and 7.04 Å, respectively. These higher variations were confined to certain areas of the structures and were not uniform. The differences in the conformational behavior of the two components were also confirmed by radius of gyration analysis (Figure 12D,E). The mean Rg for TLR7 was 33.44 Å with a range of 31.46 Å to 39.19 Å, while the mean Rg of the vaccine construct was 24.37 Å with a range of 23.61 Å to 25.70 Å. The vaccine Rg was gradually reduced and stabilized during the simulation, as there was increased compactness in the vaccine. The summary of molecular dynamics is given in Table 9 below.

The complex had an average of ~23.7 hydrogen bonds, and the instantaneous values varied between 10 and 35 along the trajectory (Figure 12G). The total solute energy has been consistently negative throughout the simulation, with a mean energy of ~−1111.5 kcal/mol and a range from −1468.9 to −721.7 kcal/mol (Figure 12F). Overall, the MD trajectories suggested the vaccine–TLR7 relationship was maintained throughout the simulated time, and there were initial conformational changes in the vaccine and retained intermolecular hydrogen-bond interactions.

### 3.19. In-Silico Immune Simulation Dynamics

**Figure 13:**
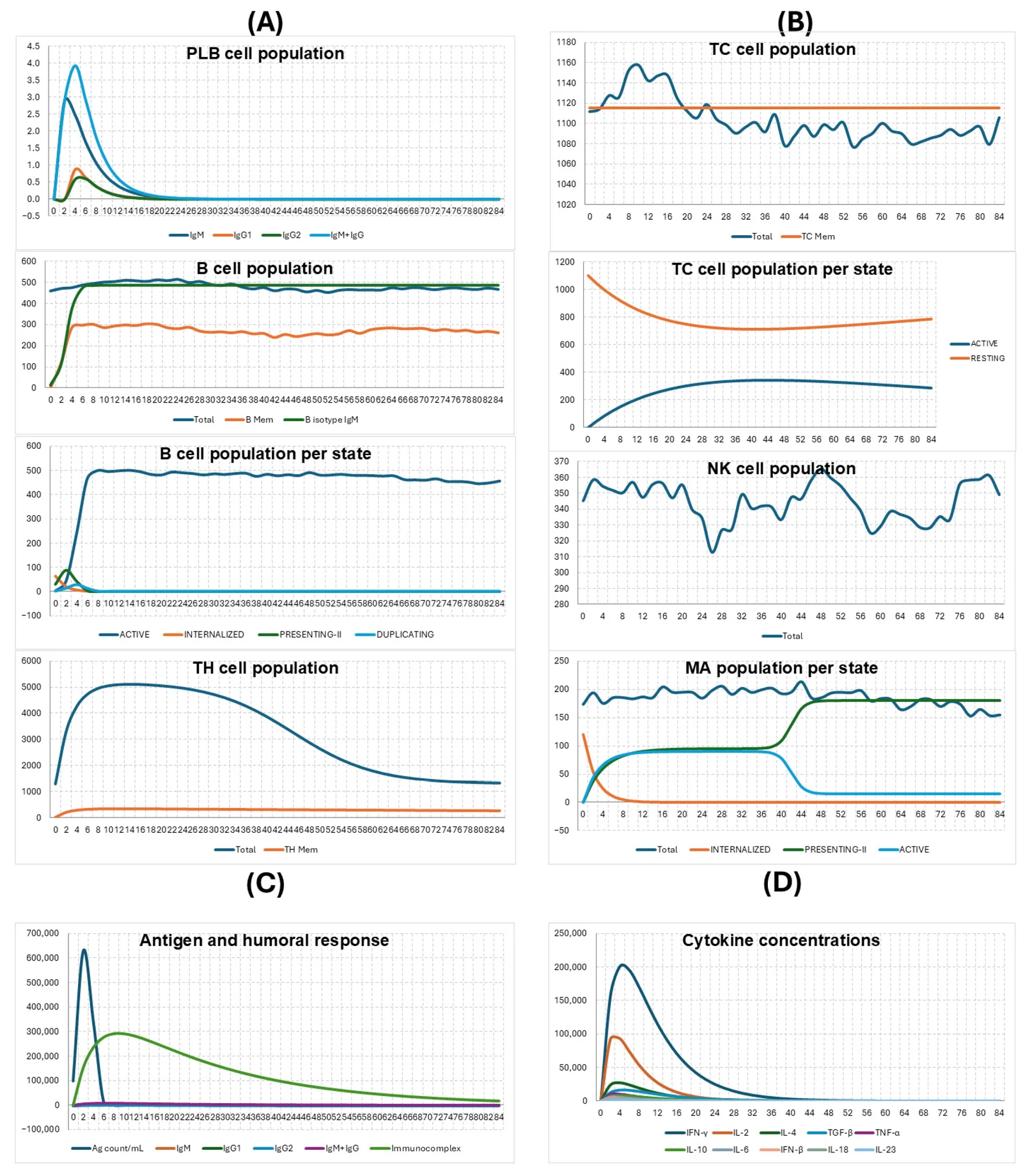
In silico immune-response simulation of the designed multi-epitope vaccine using C-ImmSim. (**A**) Predicted B-cell population dynamics, including total, active, and memory B-cell responses. (**B**) Helper T-cell population dynamics and development of activated and memory T-cell populations. (**C**) Cellular immune responses involving cytotoxic T cells and innate immune components, including NK cells, macrophages, and dendritic cells. (**D**) Predicted humoral and cytokine responses, showing antigen clearance, immunoglobulin production, and changes in cytokine concentrations during the simulation period.

The C-ImmSim analysis predicted a coordinated humoral and cellular immune response after the designed multi-epitope vaccine was administered. During the early phase of simulation, B-cell populations rapidly activated with the emergence of memory B-cell populations, indicating the establishment of a sustained humoral immune response (Figure 13A). There was significant cell stimulation of the helper T-cell populations after antigen exposure, as demonstrated by activation and memory-cell generation (Figure 13B). To further support the prediction of involvement of both adaptive and innate immune components, populations of both cytotoxic T-cells and innate immune cells (natural killer cells, macrophages, and dendritic cells) were maintained throughout the simulated period (Figure 13C).

The humoral response simulated was characterized by an increase in the production of immunoglobulins, especially those involving IgM and IgG, and a progressive decrease in the amounts of antigen over time (Figure 13D). The cytokine profiling also revealed a strong early immune response along with the presence of other cytokine signals, which were marked by the presence of IFN-γ, and a subsequent decrease along the simulated response. Taken together, these results indicate the expected ability of the vaccine construct designed to induce antibody responses, activation of cellular immunity, immune-cell memory and cytokine responses.

## 4. Discussion

In the present study, a computational pan-betacoronavirus multi-epitope vaccine platform was developed by combining evolutionary conservation, immunological screening, mutational vulnerability, structural assessment, and receptor-interaction analyses. A comparative sequence analysis of 120 spike proteins showed that there was significant conservation across the sequences even though the different betacoronavirus groups were phylogenetically separated [37,38]. But importantly, conservation was not uniform—there were actually some places that were more diverse and others that were more constrained. Based on these conserved regions, B-cell, CTL, and HTL epitopes with favorable predicted antigenicity, safety, and HLA binding were identified and determined to be highly sequence conserved. The panel of both the CTL and the HTL also demonstrated good HLA coverage in the entire world, with mutational scanning revealing positions in the selected epitopes that have either a low number of tolerated mutations or are escape-sensitive [38,39].

These epitopes were then combined in a 409-residue multi-epitope construct that contains B-cell, MHC-I, and MHC-II epitopes, linkers between epitopes, and an adjuvant molecule, β-defensin-2. The construct was predicted to have antigenicity and desirable physical/chemical properties such as an instability index that was within the range of a stable protein and near-neutral overall hydropathicity. The feasibility of the architecture built was further validated by means of structural modeling, where 99.7% of all the evaluated residues had a favorable or additional allowed Ramachandran region. There were several different vaccine-TLR7 orientations that were favorable for docking, and molecular dynamics simulations for 100 ns after the initial docking showed that the complex was stabilizing itself after conformational changes, although the vaccine was more flexible than TLR7 [40,41].

The findings indicate that integrating sequence conservation, immunological properties, and evolutionary vulnerability provides a more stringent strategy for selecting vaccine epitopes than antigenicity-based screening alone. It is noteworthy that some conserved regions of the spike have high predicted HLA binding, indicating that regions conserved evolutionarily may have immunological significance in genetically diverse human populations [42,43]. Concurrently, specific mutational scanning of conserved epitopes also revealed escape-sensitive residues that were not conserved in the present day, highlighting the importance that conservation at the present time does not imply stability in the future. Therefore, by combining conservation with mutation-resistance profiling, epitopes with higher relevance over time against newly emerged betacoronaviruses may be better selected [44,45].

The present findings are in line with the previous studies of SARS-CoV, MERS-CoV, and SARS-CoV-2 conducted by immunoinformatics approaches that identified and prioritized conserved B cell and T cell epitopes based on their antigenicity, HLA binding, population coverage, safety assessment, structural modeling, and molecular docking. But the majority of these methods have been developed for a single coronavirus species or the predominant variants found in circulation [46]. The current work further extends this approach, including the cross-betacoronavirus conservation, the screening for diversity using entropy-based approaches, and the evaluation of mutational escape prior to vaccine development. This evolution-oriented approach thus offers another level of selection to search for the epitopes which may be more immune-friendly as well as more resistant to future viral diversification [47,48,49].

The previous computational studies have suggested a multi-epitope-based coronavirus vaccine approach in which conserved B cell epitopes, CTL, and HTL epitopes were prioritized along with their evaluations with regard to antigenicity, HLA coverage, structural properties, and immune response [50]. In recent years, constructs of pan-betacoronaviruses based on conserved RBD regions of SARS-CoV, MERS-CoV, and SARS-CoV-2 were also developed. The present study is different in that it combines conservation of sequence across betacoronaviruses, phylogenetic analysis, entropy-based diversity profiling, and predicted levels of mutational escape to finalize epitope prioritization. This layer evolved from the evolution and is used as an additional criterion to distinguish candidate epitopes that have relatively less predicted sequence variation [50].

The sequence dataset was strongly biased toward SARS-CoV-2 (100/120 sequences; 83.3%), whereas other betacoronavirus lineages were comparatively underrepresented. This imbalance may influence estimates of pan-betacoronavirus conservation and limits the generalizability of the identified conserved regions. Future analyses should incorporate larger and more balanced lineage-level sampling. The Shannon entropy is used to determine areas of high sequence diversity, as well as areas of high potential for recombination, but does not directly show recombination or predict future events. Furthermore, the study is fully computational, and thus the predicted antigenicity, HLA binding, cytokine induction, population coverage, mutational robustness, receptor docking, and MD stability cannot be verified as real immunogenicity or protection efficacy. Likewise, computational interaction with TLR7 is not a sign of receptor activation but needs to be verified by experiments. TLR7 physiologically recognizes endosomal ssRNA-derived ligands and specific small-molecule agonists; therefore, the predicted vaccine–TLR7 interaction observed here should not be interpreted as evidence of direct TLR7 activation. Functional receptor activation requires experimental validation using appropriate cell-based signaling assays [51,52]. TLR7 physiologically recognizes RNA-derived ligands generated within the endolysosomal compartment rather than peptide antigens. Therefore, the predicted vaccine–TLR7 association observed by docking and MD simulation should be regarded as exploratory structural evidence only and does not demonstrate direct TLR7 activation or downstream signaling. Experimental receptor-binding and cell-based pathway activation assays are required to establish any functional relationship [53]. Human–mouse TLR7 sequence and structural differences were not evaluated in the present study and may affect the generalizability of the predicted vaccine–TLR7 interaction to murine models. Comparative receptor analysis should therefore precede interpretation of future mouse-based validation experiments.

The study offers a model for creating a pan-betacoronavirus vaccine before a new pandemic appears—conserved and evolutionarily restricted regions of the virus are identified. This could help generate a library of pre-defined candidate epitopes that could speed up a vaccine’s development if it were to be needed after a future zoonotic spillover. Future studies should be able to include an even mix of Sarbecovirus, Merbecovirus, Embecovirus, Nobecovirus, and animal reservoir viruses and then test the vaccine candidates with experiments. Further HLA binding assays, human PBMC studies, cytokine and antibody-response analysis, recombinant expression, and appropriate in vivo challenge experiments will be necessary to ascertain the potential of cross-betacoronavirus protection to translate to real-world broad and durable protection.

## 5. Conclusions

The present study underscores the potential of an evolution-oriented immunoinformatics approach for the creation of a preemptive pan-betacoronavirus vaccine, by combining spike protein conservation, immune epitope screening, population coverage, mutational vulnerability, structure modeling, receptor docking and molecular dynamics analysis. The 48 epitopes selected had good predicted immunologic and safety properties, and targeted conserved regions which are less prone to evolutionary change. These results are, however, only computed and need to be validated experimentally. Future studies should consider more balanced and larger sampling of animal and human betacoronaviruses, experimental validation of the immune response against the epitopes and cross reactivity, and testing of the construct in a suitable in vivo model. This validation has the potential to determine if this preemptive approach will offer a broad protection against existing and future Beta coronavirus pathogens.

## Figures and Tables

**Table 1:** Composition of the retrieved Betacoronavirus spike protein dataset.

Sequence Category	Number of Sequences
SARS-CoV-2	100
Bovine coronavirus	6
Human coronavirus OC43	3
Pangolin coronavirus	3
Human coronavirus HKU1	2
*Betacoronavirus* sp.	1
Records without species-level annotation in FASTA header	5
Total	120

**Table 2:** Summary of multiple sequence alignment and conservation analysis.

Parameter	Result
Spike sequences aligned	120
Alignment length	1501 positions
Ungapped protein length	1241–1363 aa
Mean residue-wise conservation	90.7%
Positions with ≥90% conservation	790
Positions with ≥95% conservation	538
Sequences represented in conserved subset	120
Conserved-subset sequence length	1023–1063 aa

**Table 3:** Summary of residue-wise spike protein conservation.

Conservation Parameter	Result
Alignment positions analysed	1501
Mean conservation score	0.915
Conservation score range	0.440–1.000
Highly conserved positions (≥0.85)	1287 (85.7%)
Positions with conservation ≥ 0.90	666 (44.4%)
Positions with conservation ≥ 0.95	587 (39.1%)
Completely conserved positions (1.00)	461 (30.7%)
Longest continuous ≥ 0.85 region	96 residues
Position of longest conserved region	1193–1288

**Table 4:** Prioritized B-cell epitopes following antigenicity, allergenicity, toxicity, and conservancy screening.

Epitope	Position	Length (aa)	VaxiJen Score	Allergenicity	Toxicity	Conservancy (%)
**IWLGFIAGLI**	1048–1059	10	0.668	Non-allergen	Non-toxic	95.5
**SFVIGEVQIAPGQT**	306–321	16	0.594	Non-allergen	Non-toxic	95.5
**GTNTSQVAVLYQV**	475–487	13	0.577	Non-allergen	Non-toxic	95.5
**TTAPAICHGKAHFP**	914–927	14	0.576	Non-allergen	Non-toxic	95.5
**RFPNITNCPFEVFNAFAS**	241–260	20	0.571	Non-allergen	Non-toxic	95.5
**WFTQRNFYEPQIITTNTFVSGNCDVVIGIVNNT**	940–972	33	0.571	Non-allergen	Non-toxic	95.5
**YNFTFKCYGVSPTKL**	280–294	15	0.565	Non-allergen	Non-toxic	95.5
**FCDPFLVHNNKYS**	98–110	13	0.556	Non-allergen	Non-toxic	—

**Table 5:** Prioritized CTL epitopes and predicted HLA class I binding.

CTL Epitope	HLA-I Allele	Predicted IC50 (nM)	VaxiJen Score	Allergenicity	Toxicity	Conservancy (%)
**FQFCDPFLV**	HLA-A∗02:06	0.939	0.5944	Non-allergen	Non-toxic	100
**EVFNAFASV**	HLA-A∗68:02	2.026	0.5556	Non-allergen	Non-toxic	100
**SQVAVLYQV**	HLA-A∗02:06	3.577	0.5944	Non-allergen	Non-toxic	100
**CPFEVFNAF**	HLA-B∗35:01	3.695	0.5556	Non-allergen	Non-toxic	100
**KIYSKHTPI**	HLA-A∗32:01	4.101	0.5167	Non-allergen	Non-toxic	100
**FPNITNCPF**	HLA-B∗35:01	4.371	0.5167	Non-allergen	Non-toxic	100
**FQFCDPFLV**	HLA-A∗02:01	5.599	0.5944	Non-allergen	Non-toxic	100
**AAYYVGYLR**	HLA-A∗31:01	8.231	0.5944	Non-allergen	Non-toxic	100
**SFRGYYPDK**	HLA-A∗30:01	9.638	0.5167	Non-allergen	Non-toxic	100

**Table 6:** Identified prioritized HTL epitopes and calculated HLA class II binding epitopes.

HTL Epitope	HLA-II Allele	Predicted IC50 (nM)	VaxiJen Score	Allergenicity	Toxicity	Conservancy (%)
**KNDGYFKIYSKHTPI**	HLA-DRB1∗11:01	28	0.52	Non-allergen	Non-toxic	100
**DLPIGIITRFQLLAL**	HLA-DPA1*02:01*/*DPB1*01:01	46	0.58	Non-allergen	Non-toxic	100
**KNDGYFKIYSKHTPI**	HLA-DRB1∗15:01	82	0.52	Non-allergen	Non-toxic	100
**QDLFLPFFSNFNDGV**	HLA-DPA1*01:03*/*DPB1*02:01	106	0.56	Non-allergen	Non-toxic	100
**LPLVSQCVNLTTLPY**	HLA-DRB1∗04:01	124	0.56	Non-allergen	Non-toxic	100
**DLPIGIITRFQLLAL**	HLA-DRB4∗01:01	124	0.58	Non-allergen	Non-toxic	100
**AAAYYVGYLRTFLLY**	HLA-DPA1*01:03*/*DPB1*02:01	133	0.60	Non-allergen	Non-toxic	100
**KPFERDISTEIYQAG**	HLA-DRB3∗01:01	178	0.50	Non-allergen	Non-toxic	100
**DLPIGIITRFQLLAL**	HLA-DRB1∗08:02	340	0.58	Non-allergen	Non-toxic	—

**Table 7:** The cytokine-induction profiles of the selected epitopes.

Epitope	Type	IFN-γ	IFN-γ Score	IL-4	IL-4 Score	IL-10	IL-10 Score
**IWLGFIAGLI**	B-cell	Positive	0.750	Positive	0.660	Negative	0.320
**SFVIGEVQIAPGQT**	B-cell	Positive	0.636	Negative	0.364	Negative	0.400
**GTNTSQVAVLYQV**	B-cell	Positive	0.596	Negative	0.438	Negative	0.358
**TTAPAICHGKAHFP**	B-cell	Positive	0.579	Negative	0.364	Negative	0.400
**RFPNITNCPFEVFNAFAS**	B-cell	Positive	0.728	Positive	0.500	Negative	0.406
**WFTQRNFYEPQIITTNTFVSGNCDVVIGIVNNT**	B-cell	Positive	0.768	Negative	0.436	Negative	0.314
**YNFTFKCYGVSPTKL**	B-cell	Positive	0.817	Positive	0.600	Negative	0.297
**FCDPFLVHNNKYS**	B-cell	Positive	0.796	Positive	0.577	Negative	0.358
**FQFCDPFLV**	MHC-I	Positive	0.817	Positive	0.700	Negative	0.328
**EVFNAFASV**	MHC-I	Negative	0.528	Positive	0.500	Positive	0.561
**SQVAVLYQV**	MHC-I	Positive	0.706	Positive	0.500	Negative	0.406
**CPFEVFNAF**	MHC-I	Negative	0.528	Positive	0.600	Negative	0.406
**KIYSKHTPI**	MHC-I	Positive	0.794	Negative	0.400	Negative	0.328
**FPNITNCPF**	MHC-I	Positive	0.617	Positive	0.500	Negative	0.250
**AAYYVGYLR**	MHC-I	Positive	0.728	Positive	0.700	Negative	0.406
**SFRGYYPDK**	MHC-I	Negative	0.528	Positive	0.600	Negative	0.406
**DLPIGIITRFQLLAL**	MHC-II	Positive	0.977	Positive	0.600	Negative	0.343
**KNDGYFKIYSKHTPI**	MHC-II	Positive	0.870	Negative	0.480	Negative	0.343
**KPFERDISTEIYQAG**	MHC-II	Positive	0.563	Negative	0.420	Negative	0.483
**LPLVSQCVNLTTLPY**	MHC-II	Positive	0.977	Positive	0.600	Negative	0.297
**QDLFLPFFSNFNDGV**	MHC-II	Positive	0.563	Positive	0.660	Negative	0.390
**AAAYYVGYLRTFLLY**	MHC-II	Positive	0.763	Positive	0.780	Negative	0.390

**Table 8:** Predicted physicochemical properties of the multi-epitope vaccine construct.

Parameter	Predicted Value
Sequence length	409 aa
Molecular weight	45,125.19 Da (~45.13 kDa)
Theoretical pI	9.39
Positively charged residues (Arg + Lys)	42
Negatively charged residues (Asp + Glu)	18
Instability index	27.16
Stability classification	Stable
Aliphatic index	72.79
GRAVY	−0.052
Estimated half-life, mammalian reticulocytes	30 h
Estimated half-life, yeast	>20 h
Estimated half-life, *E. coli*	>10 h

**Table 9:** Summary of molecular dynamics parameters for the vaccine–TLR7 complex.

MD Parameter	Vaccine	TLR7/Complex
Simulation duration	100 ns	100 ns
Mean RMSD (Å)	6.55	2.93
Maximum RMSD (Å)	7.18	3.46
Mean RMSF (Å)	2.06	1.33
Maximum RMSF (Å)	7.39	7.04
Mean radius of gyration (Å)	24.37	33.44
Rg range (Å)	23.61–25.70	31.46–39.19
Mean hydrogen bonds	—	23.68
H-bond range	—	10–35
Mean total solute energy (kcal/mol)	—	−1111.54
Total energy range (kcal/mol)	—	−1468.94 to −721.67

## Data Availability

The sequence data analyzed in this study were obtained from publicly available NCBI GenBank records. The processed datasets and supporting data generated during this study are available from the corresponding author upon reasonable request. Custom scripts used for data processing and computational analyses are available from the corresponding author upon reasonable request.
